# Understanding the functional form of the relationship between childhood cognitive ability and adult financial well-being

**DOI:** 10.1371/journal.pone.0285199

**Published:** 2023-06-07

**Authors:** Joe Gladstone, Jenna Adriana Maeve Barrett

**Affiliations:** 1 Leeds School of Business, University of Colorado, Boulder, Colorado, United States of America; 2 School of Business and Economics, Maastricht University, Maastricht, The Netherlands; The Open University, UNITED KINGDOM

## Abstract

The increasing complexity of the modern financial landscape presents significant challenges for individuals’ financial well-being. In this study, we aim to investigate the relationship between cognitive ability and financial well-being by utilizing data from the British Cohort Study, which follows a sample of 13,000 individuals from birth in 1970 to the present day. Our objective is to examine the functional form of this relationship while controlling for factors such as childhood socio-economic status and adult income. Previous research has established a correlation between cognitive ability and financial well-being, but has implicitly assumed a linear relationship. Our analyses indicate that the majority of the relationships between cognitive ability and financial variables are monotonic. However, we also observe non-monotonic relationships, particularly for credit usage, suggesting a curvilinear relationship where both lower and higher levels of cognitive ability are associated with lower levels of debt. These findings have important implications for understanding the role of cognitive ability in financial well-being and for financial education and policy, as the complexity of the modern financial landscape poses significant challenges for individuals’ financial well-being. As financial complexity is increasing and cognitive ability is a key predictor of knowledge acquisition, misspecifying the true relationship between cognitive ability and financial outcomes leads to an undervaluation of the role of cognitive ability for financial well-being.

## Introduction

The financial behaviors and outcomes of consumers exhibit significant variation. While some individuals are able to accumulate wealth and achieve financial success [1], others struggle and become financially vulnerable [2]. The increasing complexity of the financial landscape requires consumers to have a deep understanding of financial information in order to make decisions that promote their financial well-being [3]. As financial complexity continues to increase, cognitive ability emerges as a key individual difference factor in understanding the heterogeneity of financial decision-making among consumers.

Cognitive ability, broadly defined as “the ability to reason, plan, solve problems, think abstractly, comprehend complex ideas, learn quickly and learn from experience” [4], is a strong predictor of knowledge acquisition [5], which is crucial for making informed decisions in an increasingly complex financial system [3, 6]. Previous research has generally found positive associations between cognitive ability and financial well-being [e.g., 7–9]. However, most research on the relationship between cognitive ability and financial well-being has assumed a linear relationship without testing alternative functional forms. Yet, there are no *a priori* reasons why linear relationships should be expected over non-linear ones, which represent a much larger set of all possible relationships. This assumption of linearity may result in biased point estimates and incorrect conclusions if the true relationship is non-linear. For example, one study [9] found no significant association between cognitive ability and wealth and argued that individuals with low cognitive ability can achieve financial success and that higher cognitive ability is not necessarily an advantage. However, it is also possible that the association between cognitive ability and wealth is present but non-linear. Without testing the functional form of the relationship, previous research may have underestimated the importance of cognitive ability in shaping financial well-being. This has important implications for interventions aimed at improving cognitive ability in childhood, which have the potential to improve financial literacy, knowledge and financial outcomes [10, 11]. Therefore, it is crucial to examine the functional form of the relationship between cognitive ability and financial well-being to accurately capture the role of cognitive ability in shaping financial outcomes.

In addition to the theoretical implications, understanding the functional form of the relationship between cognitive ability and financial well-being has practical benefits for policymakers. Individuals with different levels of cognitive ability may have distinct strengths and challenges in achieving financial well-being. Current regulations, such as requiring banks to calculate and send appropriate credit card repayments alongside bills, aid consumers in making informed financial decisions [12]. However, if the relationship between cognitive ability and credit card debt is characterized by diminishing returns, targeted interventions aimed at reducing problematic debt for those with the lowest levels of cognitive ability would be more effective than interventions that apply to all consumers. Understanding the functional form of the relationship can help identify vulnerable groups and inform targeted policy interventions, with the goal of improving financial well-being of all individuals.

In this article, we investigate the prospective effects of childhood cognitive ability on adult financial well-being. Specifically, we examine the effects of cognitive ability on debt, wealth, and subjective financial well-being. These financial outcomes represent the accumulation of consumer financial decisions over time, capturing whether consumers overspend (through credit) or sacrifice today by saving money for the future and how these decisions translate into a subjective sense of financial well-being or distress. We use a unique longitudinal dataset, the British Cohort Study, in which cognitive ability is measured at age 10 and adult financial variables are measured over 35 years. Measuring cognitive ability in childhood allows for an accurate representation of cognitive ability, as it is relatively stable over the life course [13, 14]. By measuring cognitive ability in childhood, we are able to control for childhood and parental economic relationships, which enables us to study the relationship between childhood cognitive ability and adult financial well-being with greater precision. The main objective of this study is to determine the functional form of the relationship between cognitive ability and financial well-being. This information can help us to improve our understanding of how cognitive ability influences financial outcomes and can inform targeted interventions to improve financial well-being. One important exception is a study [9] that documents a non-linear relationship between cognitive ability and three specific negative financial behaviors (i.e., being late paying bills, maxing out a credit card, and going bankrupt). Specifically, across all three outcomes, he finds a pattern where negative financial behaviors peak for those closer to average levels of cognitive ability and are more positive for those with both higher and lower levels of cognitive ability. However, our study expands on this research by examining different financial outcomes and following the same group of individuals over their lifetimes, allowing us to use measures of cognitive ability from childhood and reduce the possibility of reverse causality.

Our second contribution is to test whether the effect of cognitive ability on adult financial outcomes is solely driven by differences in income. Given the positive relationship between cognitive ability and income [see 15, for a meta-analysis], any effect of cognitive ability on adult financial outcomes may simply reflect differences in income, as income is known to play an important role in increasing financial well-being [16]. Thus, we assess whether there is an effect of cognitive ability beyond income to gain a more accurate estimate of its unique effect on financial outcomes. Controlling for income allows us to isolate the direct relationship between cognitive ability and financial outcomes, and our results indicate that cognitive ability is indeed associated with financial well-being. This approach allows us to disentangle whether higher levels of cognitive ability are beneficial for financial well-being or whether there is no advantage of higher cognitive ability beyond its effect on income.

### Cognitive ability and financial decision-making

The positive association between cognitive ability and financial decision-making is well-established by past research. Studies have shown that higher cognitive ability is positively associated with financial outcomes, such as greater wealth [17–21], stock market participation [22, 23], and financial planning [24]. Conversely, lower cognitive ability is associated with adverse financial behavior and outcomes, such as making more financial mistakes [6], having less financial knowledge [17], being less likely to participate in market recovery [25], and being more likely to default on mortgages [26].

Research on the relationship between cognitive ability and debt accumulation is more limited and inconclusive, with mixed findings. For example, a recent laboratory experiment found that individuals with lower cognitive ability were less averse to debt [27], and a neuroscientific study [28] found that individuals with impaired executive functioning acquired more credit card debt. In contrast, another study found that for elderly individuals in the United States, those with high cognitive ability were more likely to hold debt [3], possibly due to the complexity of credit products. Debt is a complex phenomenon that can have both positive and negative outcomes. Debt can be used to smooth consumption and to make financial (e.g. mortgages) and human capital (e.g. student loans) investments [29]. At the same time, excessive debt can lead to stress and financial instability [30]. Therefore, as debt does not necessarily reflect an absence of financial well-being, in our study, we focus on forms of debt that indicate suboptimal financial decision-making and exclude mortgage debt and credit card debt that is paid off monthly.

There are two main reasons why cognitive ability may lead to more positive financial decision-making. First, cognitive ability aids the knowledge acquisition and skills needed to make informed financial choices. People with higher cognitive ability are better at accessing and processing financial information, as they have a lower cost of acquiring such information [8, 25, 31]. Additionally, cognitive ability is positively associated with financial literacy, as it helps individuals understand basic financial concepts and make informed financial decisions [11, 25]. Grasping basic financial concepts, such as the effect of compounded interest on the value of one‘s savings, is indispensable in understanding the importance of starting to save early for distant goals such as retirement. Similarly, knowledge about the costs of credit is invaluable in deciding whether to save for unexpected expenditures, such as those related to the breakdown of home appliances, or to take up credit to cover such costs. While financial literacy is essential for sound decision-making, many households lack an understanding of basic financial concepts [32]. This lack of financial literacy poses a challenge, as financial literacy has been identified as a key variable to explain variation in a household’s tendency to accumulate wealth [33]. Although it is challenging to disentangle the effects of cognitive ability and financial literacy in adulthood, cognitive ability is typically thought to precede financial literacy [10, 34–36]. As the knowledge required for good financial decision-making changes over time, general cognitive ability allows individuals to learn relevant financial skills when needed [36], and there is a large body that demonstrates the relevance of financial literacy for sound decision-making [e.g., 37, 38]. Second, the preferences of individuals with higher cognitive ability align more closely with sound financial behavior. For example, a meta-analysis [39] found that higher cognitive ability is associated with less delay-discounting. On average, individuals with higher cognitive ability are more patient [e.g., 40], which can enable them to pursue education [21], succeed in employment [41] and thus accumulate wealth. Furthermore, cognitive ability is associated with avoidance of negative risks, such as substance abuse, and approaching positive risks, such as stock market participation [42]. However, while the evidence suggests that cognitive ability is associated with skills and preferences that precede positive financial outcomes, this does not imply that increased cognitive ability monotonically increases financial well-being.

### The functional form of the relationship between cognitive ability and positive financial decision-making

**Fig 1:**
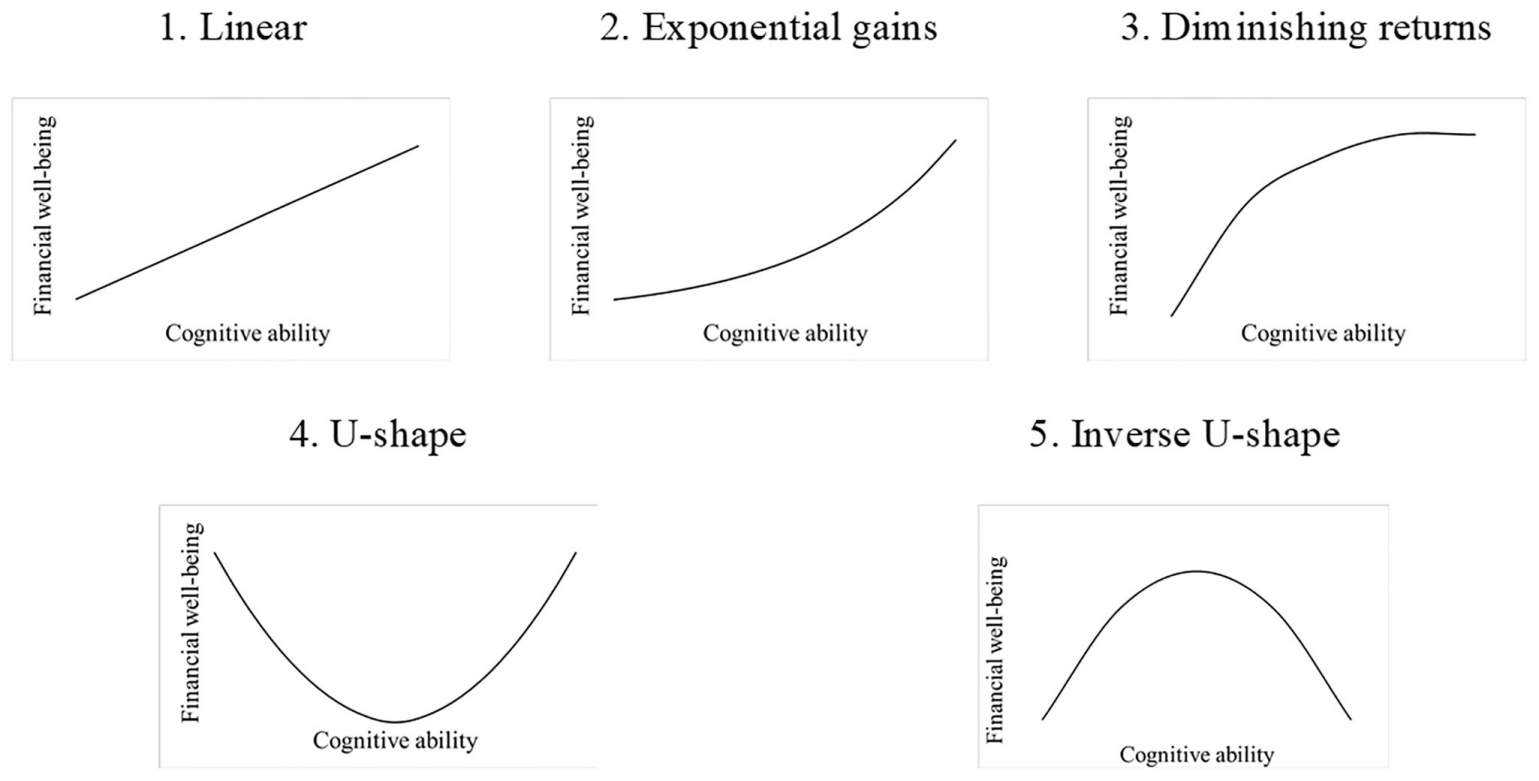
Possible functional forms of the relationship between cognitive ability and financial well-being.

The nature of the relationship between cognitive ability and financial well-being is currently unclear. We describe five possible types of relationships that may characterize the association between cognitive ability and measures of financial well-being (Fig 1).

#### Linear

Most previous literature on cognitive ability and financial well-being has modelled a positive linear relationship (Fig 1, panel 1), in which cognitive abilities are associated with more positive financial decision-making at all levels of cognitive ability [e.g., 18]. This assumption is partially based on findings in related domains, such as a meta-analysis [43], which found that non-linear relationships were only observed in 5–6% of studies on the relationship between cognitive ability and job performance. Similarly, another study [44] found that a linear relationship best fits the relationship between cognitive ability and a variety of performance measures. It is therefore reasonable to expect a linear relationship between cognitive ability and financial well-being, as increases in cognitive abilities and related skills and preferences could consistently improve a person’s financial decisions.

#### Exponential gains

The relationship between cognitive ability and financial well-being may be characterized by exponential gains (Fig 1, Panel 2). This means that a small increase in cognitive ability leads to a small increase in positive financial outcomes, but a large increase in cognitive ability results in disproportionately more positive financial outcomes. For example, individuals in the 90^th^ percentile of cognitive ability outperform others on tasks beyond what would be expected from a linear relationship [45]. Similarly, individuals in the 99^th^ percentile of cognitive ability are more likely to achieve exceptional outcomes, such as securing patents [46]. Given that cognitive ability is associated with an increased willingness to take risks and greater patience [47], the compounding effects of interest from investments and savings may result in disparities in financial outcomes between those with high versus low cognitive ability, indicating an exponential relationship.

#### Diminishing returns

The relationship between cognitive ability and financial well-being could also exhibit diminishing returns, where the benefits of increased cognitive ability decline or even cease entirely after a certain point (Fig 1, Panel 3). This hypothesis aligns with Spearman’s law of diminishing returns, which argues that past a specific level of cognitive ability, differences in cognitive ability become less predictive of performance [48–50]. Additionally, some researchers have contended that the benefits of cognitive ability reach a plateau at around an IQ score of 120 (i.e., those at the 90.9^th^ percentile or greater) in terms of socially valuable characteristics, such as creativity and academic achievement [51, 52]. In the context of financial decision-making, a minimum level of cognitive ability may be necessary to understand and process complex financial information, but other (non-cognitive) factors may become more predictive of financial well-being after this initial threshold is met.

#### Curvilinear

Finally, the relationship between cognitive ability and financial well-being may be best described as curvilinear, reflecting either a U-shaped (Fig 1, Panel 4) or inverted U-shaped relationship (Fig 1, Panel 5). The occurrence of negative behaviors, such as delinquency, peaks at average levels of cognitive ability and decreases for both individuals with high and low cognitive ability [53]. This trend can be attributed to the availability of opportunities for individuals to engage in such behaviors. In the financial context, individuals with low cognitive ability may have limited access to financial products, leading to fewer opportunities for suboptimal financial decisions, such as taking out high-interest loans. Conversely, those with high cognitive ability may have sufficient income to avoid the need for credit, as their incomes cover their expenses comfortably and thus have fewer opportunities to engage in negative financial behavior. Additionally, an inverse U-shaped pattern may be present in stock-market behavior, where individuals with low cognitive ability may miss out on higher returns due to lack of participation [22, 23], while high cognitive ability may lead to overconfidence and over-trading [54], resulting in adverse effects on returns.

In our study, we maintain an open-minded and exploratory approach toward analyzing the data. We acknowledge the possibility of both linear and non-linear relationships between cognitive ability, financial resources and financial outcomes. Linear relationships imply that changes in cognitive ability or financial resources are proportional to changes in financial outcomes, while non-linear relationships suggest that these variables have a more complex effect on financial outcomes. Our aim is to avoid making assumptions and, instead, objectively examine the data to determine the nature of the relationships that exist. A comprehensive understanding of these relationships can inform future research and policy decisions.

## Method

### Dataset

The study utilizes data from the 1970 British Cohort Study (BCS70), which follows the lives of individuals born in England, Scotland, and Wales in a single week of 1970. The BCS70 has collected a wide range of information on cognitive ability and economic circumstances throughout the cohort members’ lives. The British Cohort Study (BCS70) datasets and documentation are available through the UK Data Service (beta.ukdataservice.ac.uk/datacatalogue/series/series?id = 200001). For our analyses, we combine three datasets from the GN 33229 series. The data and analysis code used in the study can be accessed at https://doi.org/10.17605/OSF.IO/8F6UV.

#### Cognitive ability

**Fig 2:**
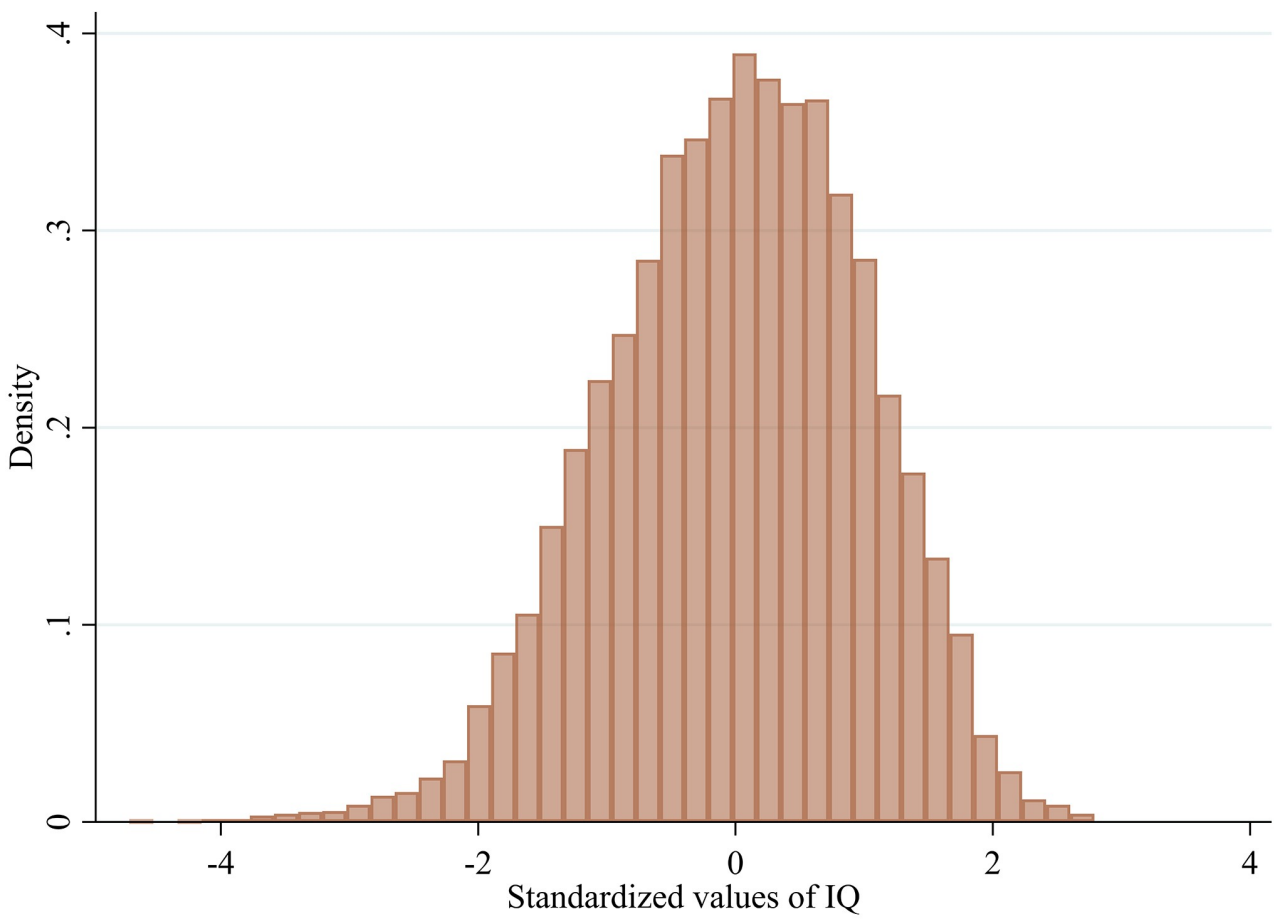
Histogram of cognitive ability scores at age 10.

The British Cohort Study collected cognitive ability data by administering eight tests to participants at age 10 [55]. These tests included the Shortened Edinburgh Reading Test [56], the Friendly Maths Test [55], the Pictorial Language Comprehension Test [55], the Spelling Dictation task [55], and four subscales of the British Ability Scales: Word Definitions, Word Similarities, Recall of Digits and Matrices [57]. A total of 12,885 participants completed at least one of the assessments, and 11,134 completed all eight [55]. Factor analysis was used to identify a general cognitive ability factor, which was used as a measure of cognitive ability in this study [55, 58–60]. This factor, known as "g," accounted for 56% of the variance in initial eigenvalues, and all tests loaded positively on the component. The cognitive ability scores were standardized and followed a normal distribution, as shown in Fig 2.

### Participants

Our study utilizes data from the 2016 wave of the British Cohort Study and includes participants who have complete measures for cognitive ability and financial outcomes. A total of 5858 cohort members (48.33% of whom are male) were included in the sample. Table 1 presents an overview of the sample characteristics, and Table 2 shows the correlations between the measures used in the study. We chose to use data from the 2016 wave of the BCS because it is the most recent available. However, we also conducted additional analyses using data from the 2012 wave of the BCS to ensure the robustness of our findings (Web Appendix A in S1 File).

**Table 1:** Descriptive statistics of financial well-being measures, as well as childhood and adult covariates.

Variable	*M* (*SD*) / %	*p25*	*Median*	*p75*
Cognitive ability	0.19 (0.95)	-0.04	0.25	0.87
*Financial well-being measures*				
Total Debt	£5342.62 (9185.90)	£0.00	£900.00	£7000
Debt-to-income Ratio	0.26 (0.83)	0.00	0.03	0.20
Presence of Credit or Store Card Debt	39.60%	-	-	-
Savings	£36,926.63 (100,044.10)	£500.00	£6000.00	£28,000.00
Asset-to-income Ratio	6.85 (157.76)	0.02	0.19	0.75
Pension Plan Membership	76.75%	-	-	-
Investment Account	29.11%	-	-	-
Subjective Financial Well-being (Financial distress)	1.93 (0.92)	1.00	2.00	2.00
*Childhood Covariates*				
Mother’s Age Left Education	16.06 (1.95)	15.00	16.00	16.00
Father’s Age Left Education	15.74 (2.10)	15.00	15.00	16.00
Family Income at Age 10	4.10 (1.22)	3.00	4.00	5.00
*Adult Covariates*				
Household Income	£42,311.41 (37,054.78)	£19,200.00	£36,000.00	£54,000.00
Household Size	3.20 (1.29)	2.00	3.00	4.00
Married	62.84%	-	-	-

*Note*. Financial variables were measured when participants were aged 46, except for pension plan membership, which was measured at age 42.

**Table 2:** Correlation table of financial well-being measures as well as childhood and adult covariates.

**Variable**	**1**	**2**	**3**	**4**	**5**	**6**	**7**	**8**	**9**	**10**	**11**	**12**	**13**	**14**
1. CA	-													
2. Debt	.07***	-												
3. Debt-to-income Ratio	.01	.45***	-											
4. CC or SC Debt	.05***	.31***	.18***	—										
5. Savings	.13***	.01	-.04**	-.11***	-									
6. Asset-to-income Ratio	.08***	-.02*	.16***	-.08***	.48***	-								
7. Pension Membership	.23***	.05***	.00	.06***	.07***	.05***	-							
8. Investment Account	.22***	.00	-.04**	-.05***	.30***	.17***	.21***	-						
9. Financial Distress	-.15***	.08***	.12***	.13***	-.23***	-.12***	-.20***	-.27***	-					
10. Mother’s Education	.19***	.04**	.02	-.02	.08***	.06***	.06*	.10***	-.03*	-				
11. Father’s Education	.25***	.03**	.03*	-.01	.10***	.10***	.08***	.12***	-.06***	.49***	-			
12. Family Income Age 10	.26***	.04***	.00	.03*	.12***	.07***	.14***	.15***	-.11***	.22***	.28***	-		
13. HH Income	.21***	.13***	-.19***	-.01	.38***	-.07***	.16***	.28***	-.33***	.09***	.12***	.19***	-	
14. HH Size	.05***	.09***	-.01	.06***	.06***	-.02	.06***	.09***	-.06***	.06***	.06***	.06***	.19***	-
15. Married	.19***	.09***	-.02	.06***	.08***	.01	.18***	.16***	-.20***	.05***	.06***	.10***	.25***	.43***

### Measures of financial well-being

#### Household debt usage

To assess financial well-being, we employed a variety of measures. Debt usage was measured in three ways: total debt, debt-to-income ratio, and revolving credit card usage. In the British Cohort Study, respondents were asked to indicate the types of debt they or their partner had. This measure included a variety of debt sources, such as credit card debt, personal loans and catalogue or mail order purchases. Respondents were asked to exclude mortgage debt and credit cards and bills that would be paid off fully in the current month. Respondents were then asked to report their total amount of debt. The total debt measure was collected on a continuous scale, and outliers were removed by winsorizing the measure at the 1st and 99th percentiles. The average household debt of respondents in our sample at age 46 was £5342.62 (*SD* = 9185.90).

We also created a debt-to-income ratio by dividing the amount of debt by household income. A high debt-to-income ratio indicates that individuals may face difficulties in paying off their debts and making regular loan payments. In our sample, the average debt-to-income ratio was 0.26 (SD = 0.83), meaning that for every £1 of annual income, the individual owes 26 pence in debt.

Furthermore, we developed a binary variable to reflect whether respondents had revolving credit card debt, which refers to credit card debt that will not be paid off in full by the end of the month. The British Cohort Study elicits credit card debt and store card debt as separate categories, but as store cards are a specific type of credit card (i.e., a credit card that can only be used to make purchases at a specific store or chain of stores), we merge these into a single category for analyses. Revolving credit card debt is considered problematic, as it may lead to overspending and subsequent over-indebtedness or bankruptcy [29]. Our results showed that 39.6% of respondents in our sample had credit card or store card debt at age 46.

#### Household savings and investments

In addition to the debt usage measures, we also assessed participants’ level of savings. Using multiple measures of savings provides a more comprehensive understanding of an individual’s financial situation. Therefore, savings and investments were measured in four ways: total household savings, asset-to-income ratio, retirement plan membership, and investment account ownership. Total savings were measured continuously by self-report and the average level of savings among our sample was £36,926.63 (SD = 100,044.10). We winsorized this variable at the 1^st^ and 99^th^ percentile in our analyses to remove outliers.

To examine the relationship between a household’s wealth and income, we calculated the asset-to-income ratio by dividing total savings by annual household income. The average ratio of total savings to annual income was 6.85 (SD = 157.76), indicating that the total savings of a household were, on average, almost seven times greater than the annual income of the household.

To measure participants’ retirement plan membership, they were asked about their participation in various types of pension plans. If they indicated that they were a member of any of these plans, their response was coded as 1. Conversely, if they indicated that they were not a member, their response was coded as 0. We found that 76.75% of the participants in the sample were members of a pension plan.

Additionally, participants indicated whether they held a popular type of tax-free investment account called a Stocks and Shares ISA. They could also indicate they had a Personal Equity Plan (PEPs), which was a similar type of scheme replaced in 1999 by ISAs. 29.11% of participants indicated that they held one of these accounts.

#### Subjective financial well-being

Subjective financial well-being was measured at age 46 by the question: “How well [respondent] is managing financially” on the following scale: 1 = Living comfortably, 2 = Doing all right, 3 = Just about getting by, 4 = Finding it quite difficult, 5 = Finding it very difficult (*M* = 1.93, *SD* = 0.92). Higher scores indicate greater levels of financial stress. We standardize this measure for the analyses.

### Childhood covariates

#### Mother’s and father’s education

At birth, the age that the mother and father were when they left education was recorded. (*M*_mother_ = 16.06, *SD*_mother_ = 1.95; *M*_father_ = 15.74, *SD*_father_ = 2.10).

#### Family income at age 10

In 1980, when participants were aged 10, the survey recorded their family income completed by the parents. These were recorded as weekly amounts in 7 categories: less than £35; £35 –£49; £50 –£99; £100 - £149; £150 - £199; £200 - £249; £250 or more. Respondents who indicated that they did not know the amount or who did not answer the question were set to the average.

### Adult covariates

#### Income

The average household income of participants at age 46 was £19,992.59 (*SD* = 33,578.82). The income measure was collected on a continuous scale, and outliers were removed by winsorizing the measure at the 1st and 99th percentiles. We present the results in units of £1000 to ease interpretation.

#### Household size

Household size was measured at age 46 (M = 3.20, SD = 1.29).

#### Marital status

Marital status was recorded at age 46. Those who indicated that they were legally married or in a civil partnership were coded as 1. Those with other forms of relationship status (single, legally separated, divorced, widowed, etc.) were coded as 0. Of the sample, 62.8% reported being married or in a civil partnership.

### Analyses

**Fig 3:**
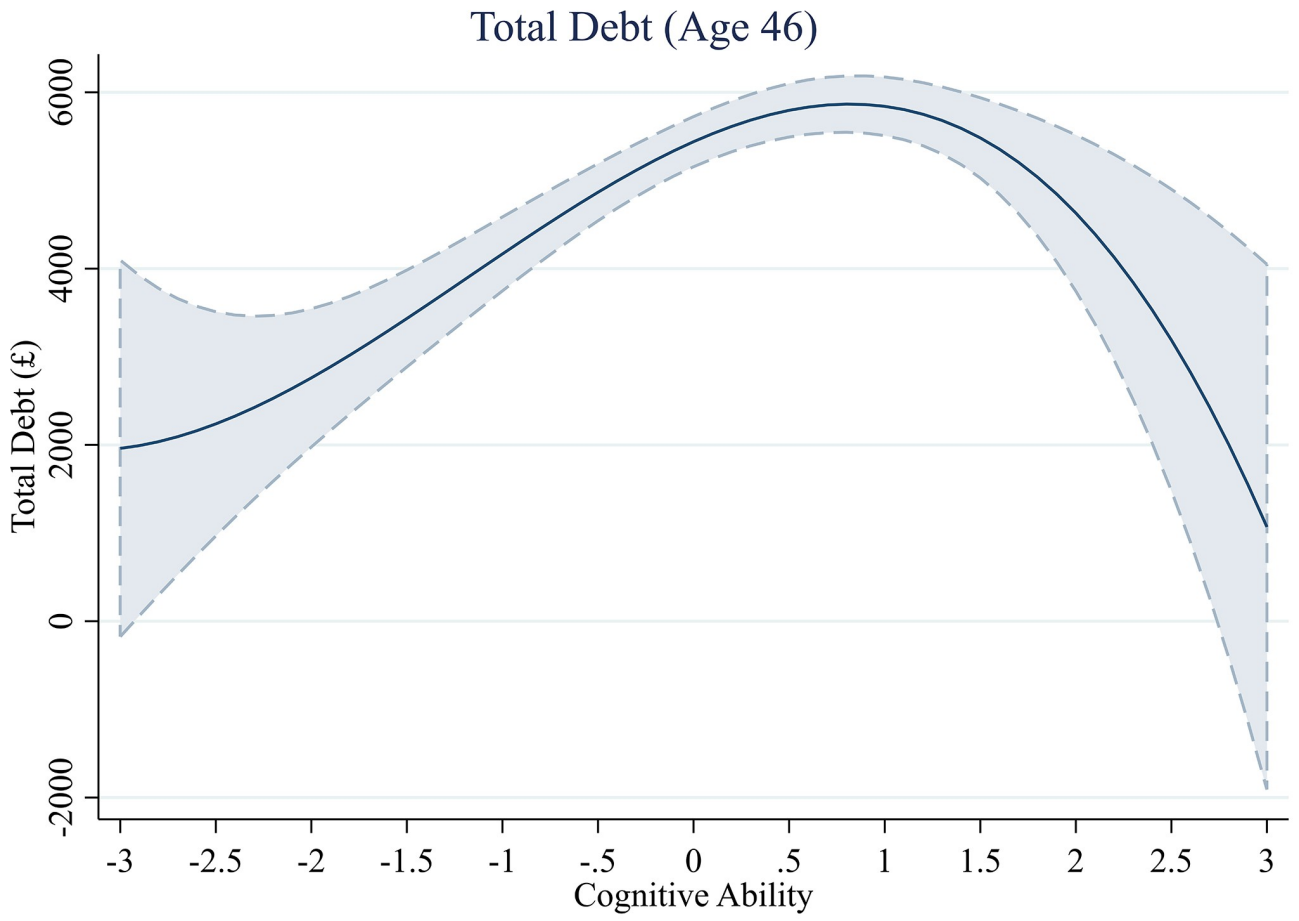
Total debt across levels of cognitive ability.

**Fig 4:**
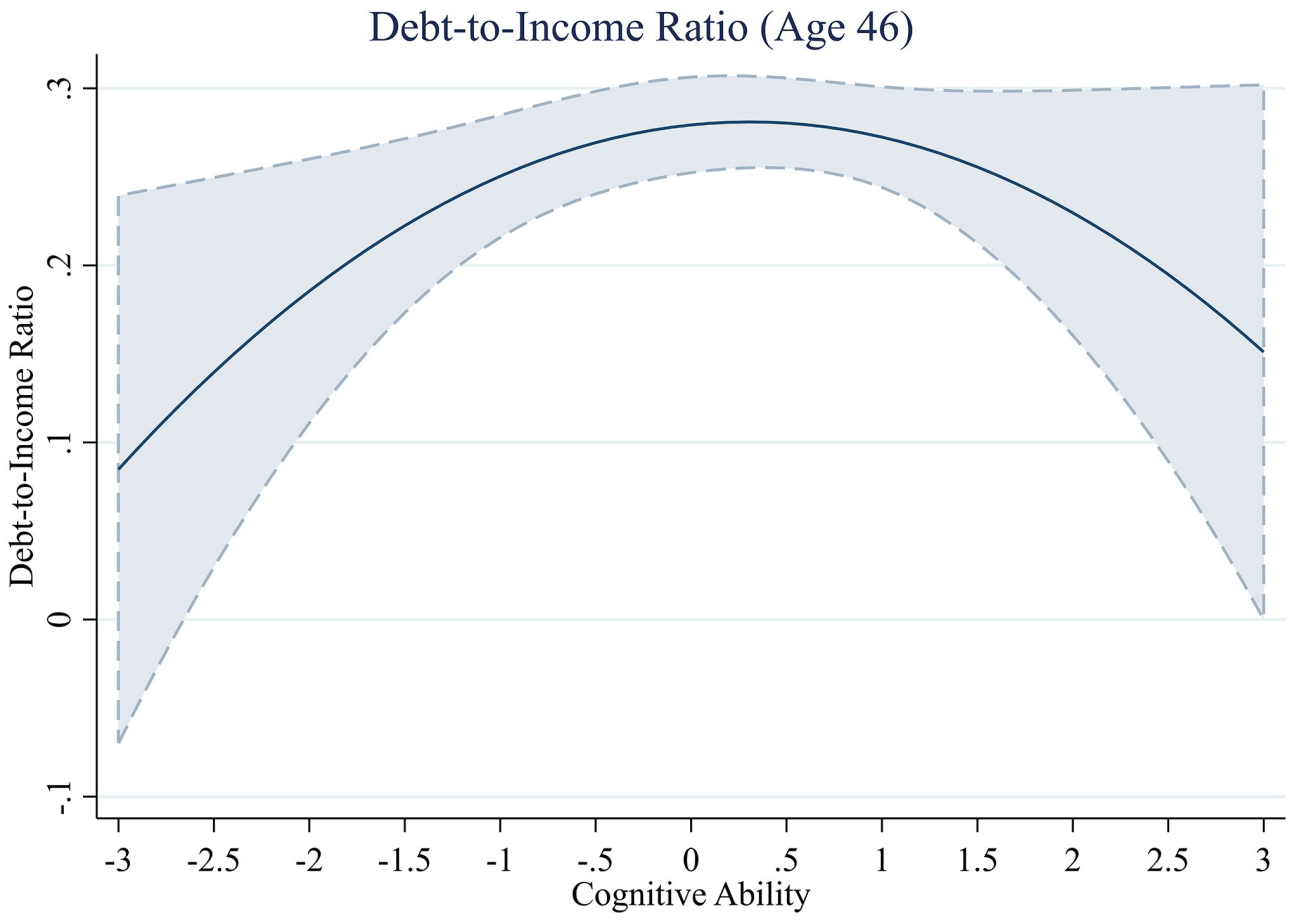
Debt-to-income ratio across levels of cognitive ability.

**Fig 5:**
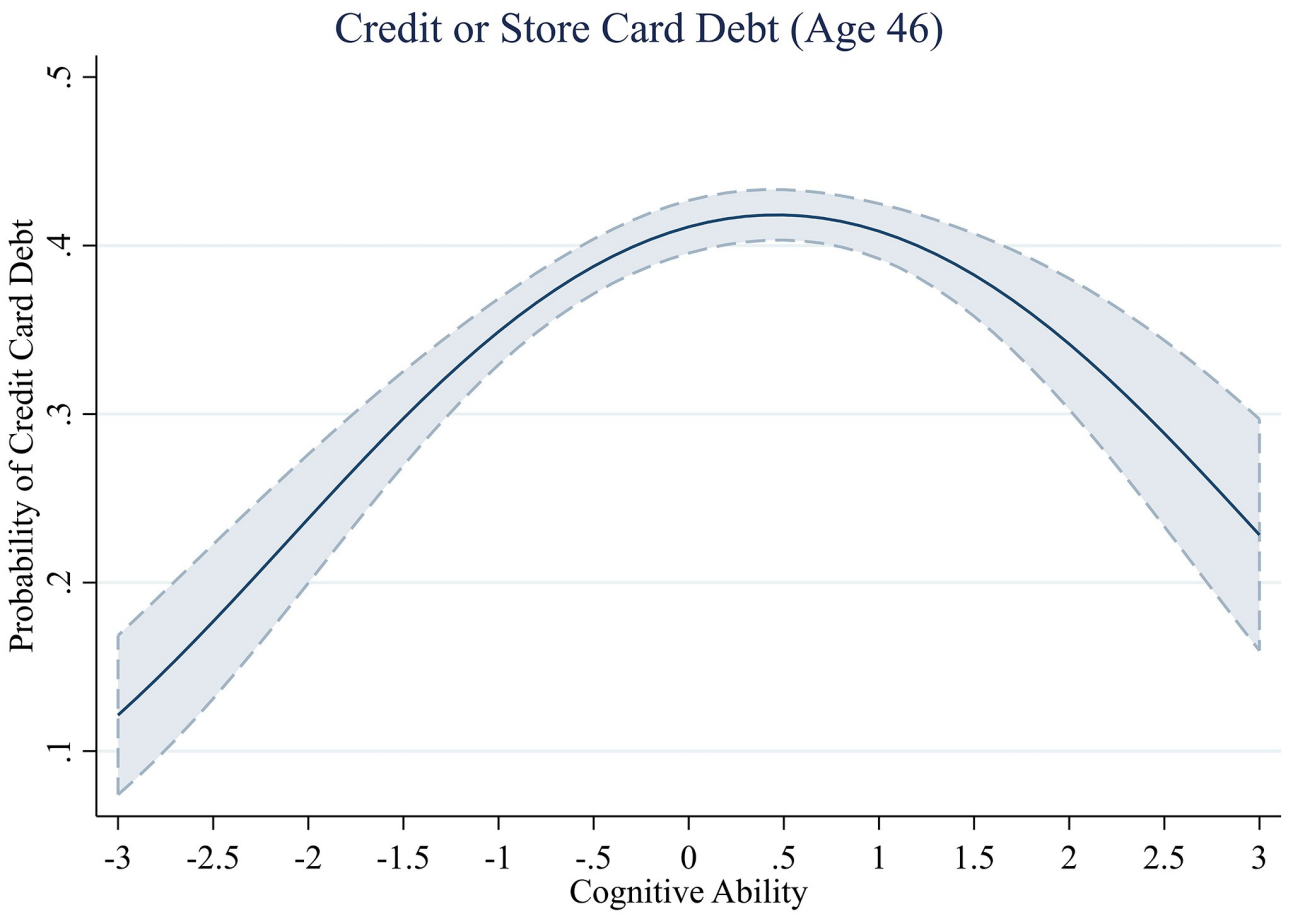
Proportion of those who hold credit or store card debt across levels of cognitive ability.

**Fig 6:**
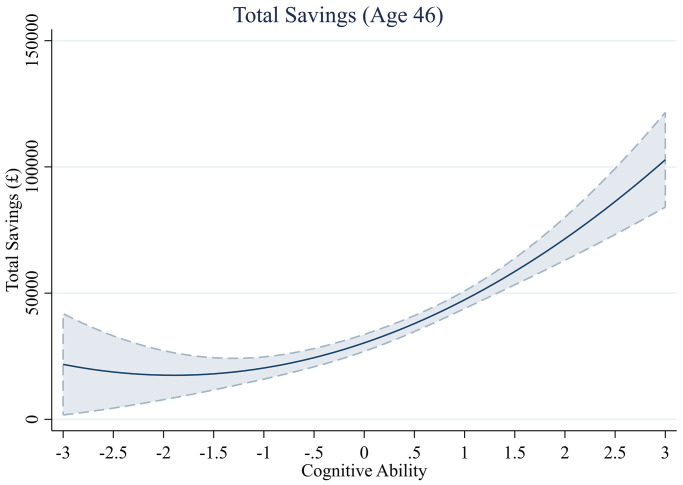
Total savings across levels of cognitive ability.

**Fig 7:**
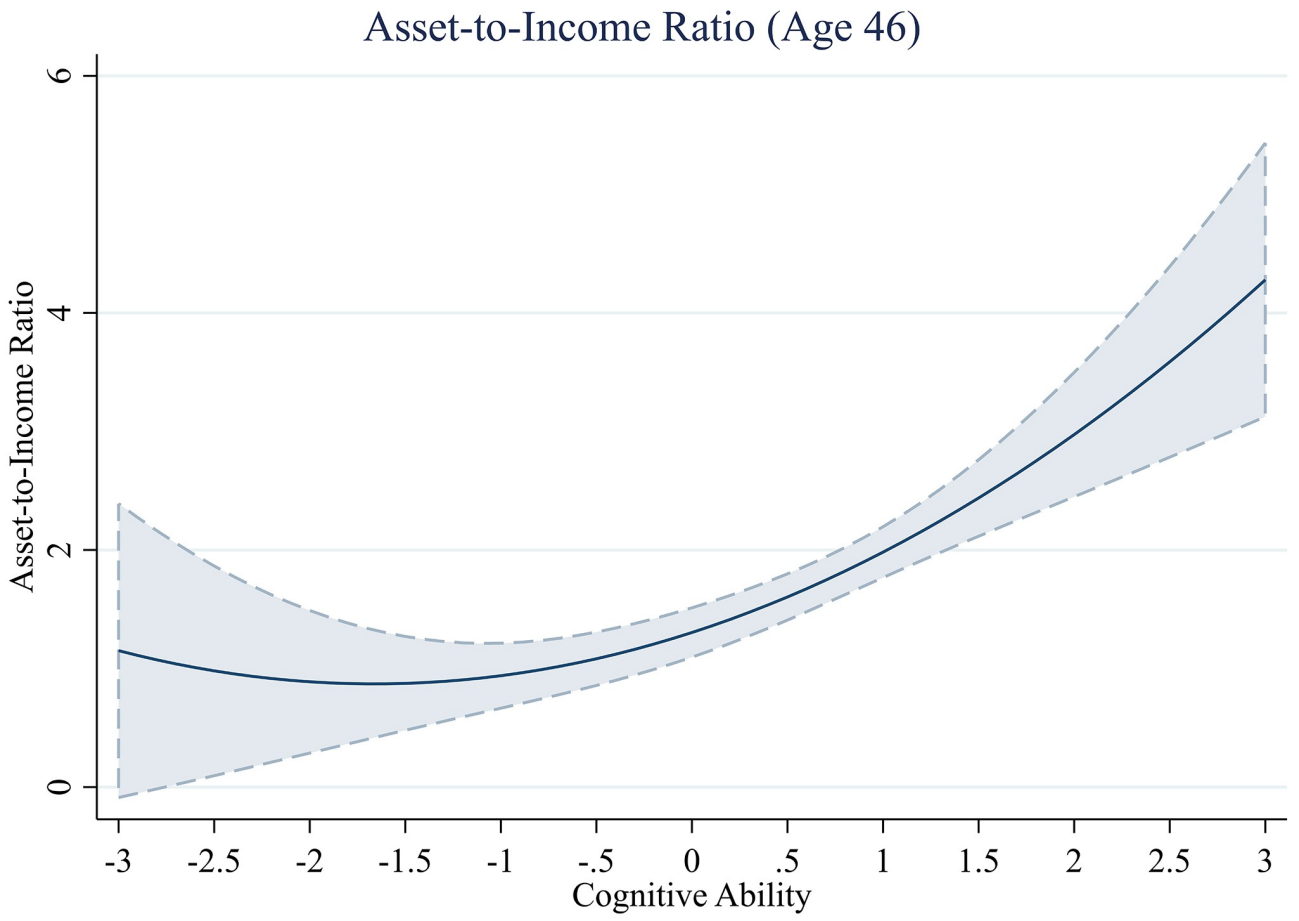
Asset-to-income across levels of cognitive ability.

**Fig 8:**
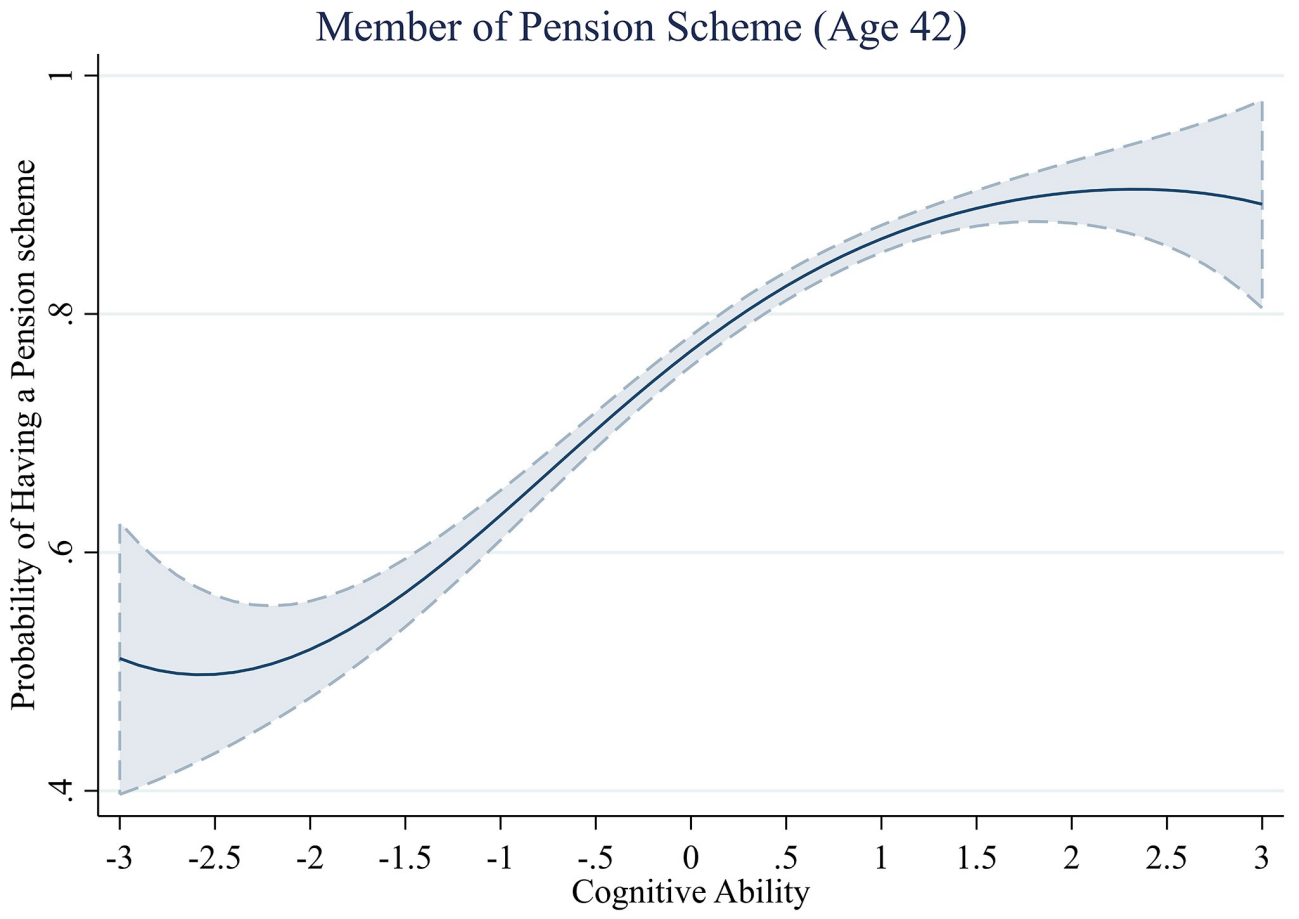
Probability of pension membership across levels of cognitive ability.

Our main interest is to investigate whether childhood cognitive ability affects later financial outcomes and to understand the shape of this relationship. To appropriately characterize the relationship between childhood cognitive ability and adult financial well-being (e.g., as linear or curvilinear), we employed polynomial models using a backward elimination procedure. We entered cognitive ability as a cubic term, as well as a quadratic and linear term, in all models, and then deleted the highest-order term one at a time until the highest-order remaining term had a significant *t*-statistic. It is recommended to keep the degree of a polynomial as low as possible and to avoid using high-order polynomials unless they can be justified for reasons outside the data [61]. As interpreting polynomial terms is complex, we plot the predicted value of financial outcomes across cognitive ability in Figs 3–8, to provide a visualization of the relationship including 95% confidence intervals. These figures depict the shape of this relationship without the inclusion of covariates.

We also aimed to determine whether the effect of childhood cognitive ability on adult financial outcomes was solely driven by income. As income is positively correlated with cognitive ability [15], differences in income may account for any association between cognitive ability and financial well-being. Given the crucial role of income in enhancing financial well-being [16], we run an additional regression model to assess the unique effect of cognitive ability beyond income. This approach allows for a more accurate estimation of the independent effect of cognitive ability on financial outcomes.

Our selection of covariates was based on prior research indicating their association with financial well-being, as well as their potential confounding effect on the relationship between cognitive ability and financial well-being. Specifically, we included childhood socio-economic status variables in our models–family income at age 10, father’s education, and mother’s education–based on research showing that the impact of cognitive ability on financial outcomes may be biased upward without controlling for parental circumstances [23]. We included adult covariates–gender, marital status, and household size–because these have been consistently associated with financial well-being [16, 62–65]. By including these covariates, we aim to reduce potential confounding effects and improve the precision and accuracy of our estimates of the relationship between cognitive ability and financial well-being.

We chose not to control for adult education or employment in our analyses because these variables may be considered intermediate variables or part of the causal mechanism that links cognitive ability with financial outcomes later in life. That is, education and employment may be viewed as the mechanisms through which cognitive ability affects financial outcomes. By controlling for these variables, we may be inadvertently removing the effect of cognitive ability on financial outcomes. Our primary aim was to assess the direct effect of cognitive ability on financial outcomes by excluding variables that may act as mediators in the causal pathway. We also do not control for age, as the cohort study includes participants born in the same week of the year 1970, and therefore all participants were the same age at each time point.

We split the results into three groups: debt usage, savings and wealth and subjective perceptions of finances.

## Results

### Debt usage

To investigate the relationship between cognitive ability and debt, we use the total amount of debt participants held at age 46, their total debt as a ratio of household income, and whether they held revolving debt on a credit card.

#### Total debt

We present our first set of analyses in Table 3. We find that the relationship between cognitive ability and debt follows a cubic polynomial form, with the cubic term Cognitive Ability^3^ significantly predicting total debt in Model 1 and 2 (Model 1—without controls, *b*^*3*^ = -123.22, *se* = 52.83, *t*(6,147) = -2.33, *p* = .020, Model 2 –with adult controls, *b*^*3*^ = -130.36, *se* = 52.49, *t*(6,144) = -2.48, *p* = .013). After controlling for childhood covariates and income, we no longer find a significant cubic term (*b*^*3*^ = -113.06, *se* = 68.74, *t*(4,357) = -1.64, *p* = .100) but we do find that the squared term of cognitive ability predicts total debt (Model 4 –all controls, *b*^*2*^ = -529.94, *se* = 121.09, *t*(4,357) = -4.38, *p* < .001).

**Table 3:** OLS regression model for the relationship between cognitive ability and debt usage.

VARIABLES	(1)	(2)	(3)	(4)
	Total debt (Age 46)	Total debt (Age 46)	Total debt (Age 46)	Total debt (Age 46)
Cognitive ability	960.41***	876.23***	796.87***	634.30**
	(188.58)	(188.00)	(232.37)	(243.70)
Cognitive ability^2^	-436.13***	-462.59***	-501.05***	-529.94***
	(96.53)	(96.04)	(115.45)	(121.09)
Cognitive ability^3^	-123.22*	-130.36*	-117.60	-113.06
	(52.83)	(52.50)	(66.35)	(68.74)
Female		-1,113.53***	-1,310.46***	-1,196.78***
		(229.52)	(268.54)	(280.91)
Married		990.79***	1,242.92***	986.51**
		(262.12)	(305.79)	(325.45)
Household size		471.99***	412.92***	355.45**
		(98.34)	(115.60)	(121.30)
Father’s education			1.10	4.59
			(74.48)	(79.37)
Mother’s education			165.07*	176.72*
			(77.90)	(82.09)
Family income at age 10			-1.90	-105.06
			(119.20)	(125.67)
Household income (Per £1000)				23.96***
				(3.90)
Constant	5,440.09***	3,933.04***	1,581.89	1,252.70
	(145.03)	(340.35)	(1,284.55)	(1,360.71)
Observations	6,151	6,151	4,666	4,368
*R* ^*2*^	.008	.021	.024	.032

Standard errors in parentheses

*** *p* < .001,

** *p* < .01,

* *p* < .05

We plot the model predicted levels of debt across cognitive ability in Fig 3 to assess the shape of this relationship. The figure shows a non-linear pattern: an inverted U-shape. At lower levels and higher levels of cognitive ability, people hold lower amounts of debt. However, a significant quadratic or cubic term in our regression is necessary, but not sufficient evidence for an inverted U-shaped relationship [66, 67]. We follow the advocated approach [67] to test for the presence of a U-shaped relationship by running two separate linear regressions on the data, one for low values of cognitive ability and one for high values, and verifying that one slope is positive and the other negative. The highest level of debt is held by those with a cognitive ability score 0.8 SDs above the mean (£5,866.21 in debt). We split the data at this point and run separate linear regression models on these two samples. The effect of cognitive ability on debt is positive and significant for those with cognitive ability levels below.8 (*b* = 1061.21, *se* = 175.37, *t*(4,463)) = 6.05, *p* < .001) and the effect is negative and significant for those with cognitive ability levels above 0.8 (*b* = -1321.56, *se* = 651.45, *t*(1,684)) = -2.03, *p* = .043). This supports our proposition of an inverted U-shaped relationship.

#### Debt-to-income ratio

Next, we test the relationship between cognitive ability and a household’s debt-to-income ratio and present these results in Table 4. As the dependent variable includes a measure of income, we do not run our fourth model, which includes income as a covariate. Initial analyses demonstrate that the cubic term Cognitive Ability^3^ does not predict the debt-to-income ratio (*b*^*3*^ = -00.00, *se* = 0.00, *t*(5,689) = -0.12, *p* = .903). However, we find that the quadratic term of cognitive ability significantly predicts the debt-to-income ratio, and this result is consistent with our findings for total debt (Model 1 –without controls, Model 1 –without controls, *b*^*2*^ = -0.02, *se* = 0.01, *t*(5,690) = -2.10, *p* = .036, Model 3 –childhood and adult controls, *b*^*2*^ = -0.03, *se* = 0.01, *t*(4,326) = -2.37, *p* = .018). Although the relationship between cognitive ability and debt-to-income ratio follows an inverse U-shape, it is difficult to determine the exact functional form due to the large confidence intervals at the extreme levels of cognitive ability (Fig 4).

**Table 4:** OLS regression model for relationship between cognitive ability and debt-to-income ratio.

VARIABLES	(1)	(2)	(3)
	Debt-to-income ratio (Age 46)	Debt-to-income ratio (Age 46)	Debt-to-income ratio (Age 46)
Cognitive ability	0.01	0.01	0.01
	(0.01)	(0.01)	(0.02)
Cognitive ability^2^	-0.02*	-0.02*	-0.03*
	(0.01)	(0.01)	(0.01)
Cognitive ability^3^	-	-	-
Female		-0.02	-0.02
		(0.02)	(0.03)
Married		-0.03	-0.02
		(0.03)	(0.03)
Household size		0.00	-0.00
		(0.01)	(0.01)
Father’s education			0.02*
			(0.01)
Mother’s education			-0.00
			(0.01)
Family income at age 10			-0.01
			(0.01)
Household income (Per £1000)			
Constant	0.28***	0.31***	0.13
	(0.01)	(0.03)	(0.12)
Observations	5,693	5,693	4,335
R-squared	.001	.001	.003

Standard errors in parentheses

*** p<0.001,

** p<0.01,

* p<0.05

Using the advocated approach [67] to test for the presence of a U-shaped relationship supports the notion that the evidence for an inverse U-shape relationship is inconclusive. We find that the highest debt-to-income ratio is found at a cognitive ability score of 0.4 SDs above the mean. We conduct separate linear regression models on the data split at this point and find that the effect of cognitive ability on the debt-to-income ratio is positive but not significant for those with cognitive ability levels below 0.4 (*b* = 0.04, *se* = 0.02, *t*(3,180)) = 1.77, *p* = .078), and the negative effect of cognitive ability on the debt-to-income ratio for those with cognitive ability levels above 0.4 is also not significant (*b* = -0.05, *se* = 0.04, *t*(2,510)) = -1.28, *p* = .199).

#### Credit card debt

We next investigated the relationship between cognitive ability and whether participants held revolving credit card or store card debt. As the outcome is binary, we use logistic regression models and present these analyses in Table 5. We first tested the cubic term for cognitive ability and found that, while close, the coefficient did not reach the 5% threshold of statistical significance (*b*^*3*^ = -0.03, *se* = 0.01, *z* = -1.95, *p* = .051). Therefore, we tested the squared term in the reported models. We find that in each model specification, the relationship between cognitive ability and debt follows a quadratic polynomial form, with the squared term Cognitive Ability^2^ significantly predicting the outcome in these models (Model 1 –without controls, *b*^*2*^ = -0.14, *se* = 0.02, *z* = -5.85, *p* < .001; Model 4 –all controls, *b*^*2*^ = -0.15, *se* = 0.03, *z* = -4.98, *p* < .001).

**Table 5:** Logistic regression model for the relationship between cognitive ability on having credit or store card debt.

VARIABLES	(1)	(2)	(3)	(4)
	Credit debt (Age 46)	Credit debt (Age 46)	Credit debt (Age 46)	Credit debt (Age 46)
Cognitive ability	0.13***	0.11***	0.11**	0.12**
	(0.03)	(0.03)	(0.04)	(0.04)
Cognitive ability^2^	-0.14***	-0.14***	-0.16***	-0.15***
	(0.02)	(0.02)	(0.03)	(0.03)
Cognitive ability^3^	-	-	-	-
Female		0.03	-0.01	-0.01
		(0.05)	(0.06)	(0.06)
Married		0.20**	0.23**	0.26***
		(0.06)	(0.07)	(0.07)
Household size		0.07**	0.08**	0.09***
		(0.02)	(0.03)	(0.03)
Father’s education			-0.03	-0.03
			(0.02)	(0.02)
Mother’s education			0.00	0.00
			(0.02)	(0.02)
Family income at age 10			0.01	0.01
			(0.03)	(0.04)
Household income (Per £1000)				-0.00
				(0.00)
Constant	-0.36***	-0.71***	-0.39	-0.37
	(0.03)	(0.08)	(0.29)	(0.31)
Observations	6,328	6,328	4,796	4,450
Pseudo *R*^2^	.006	.011	.011	.013

We plot the probability of holding credit or store card debt across cognitive ability in Fig 5 to assess the shape of this relationship. We see a similar U-shaped pattern as we found for total debt. Participants were less likely to hold revolving credit card debt when they were either low or high in cognitive ability, and more likely at more average levels of cognitive ability. Specifically, the model estimates that someone with a cognitive ability score of 2 SDs below the mean would have a 23.8% probability of holding credit or store card debt, while the average person (0 SD score) has a 41.1% probability and someone high in cognitive ability (+ 2 SD) a 34.2% probability.

The highest level of predicted credit card debt is for those with cognitive ability scores of 0.5, which is again similar to the 0.8 level found for total debt. Repeating the advocated approach to test for U-shaped relationships [67] again provides evidence in favour of an inverted U-shaped relationship (< 0.5, *b* = .08, *se* = 0.01, *t*(3,861)) = 6.80, *p* < .001; = > 0.5, *b* = -0.07, *se* = 0.02, *t*(2,463)) = -3.01, *p* = .003).

### Savings and wealth results

#### Total savings

We tested whether cognitive ability significantly predicts the amount of savings participants accumulated at age 46 and present these analyses in Table 6. We first tested the cubic term for cognitive ability, which was NS (*b*^*3*^ = 337.12, *se* = 639.74, *t*(5,337) = - 0.53, *p* = .598). Therefore, we tested the squared term in the reported models. We find that in the first two model specifications, the relationship between cognitive ability and savings follows a quadratic polynomial form, with the squared term Cognitive Ability^2^ significantly predicting savings in these models (e.g., Model 1—without controls, *b*^*2*^ = 3557.47, *se* = 1085.31, *t*(5,338) = 3.28, *p* = .001). However, after controlling for childhood socioeconomic status and adult income, the squared term was no longer statistically significant (e.g., Model 4—all controls, *b*^*2*^ = 2,301.53, *se* = 1,307.24, *t*(3,884) = 1.76, *p* = .078).

**Table 6:** OLS regression models for the relationship between cognitive ability and total savings.

VARIABLES	(1)	(2)	(3)	(4)
	Total savings (Age 46)	Total savings (Age 46)	Total savings (Age 46)	Total savings (Age 46)
Cognitive ability	13,506.97***	12,875.73***	11,307.20***	6,232.01***
	(1,454.71)	(1,457.22)	(1,842.84)	(1,798.54)
Cognitive ability^2^	3,557.47**	3,326.91**	3,179.35*	2,301.53
	(1,085.31)	(1,084.35)	(1,353.84)	(1,307.24)
Cognitive ability^3^	-	-	-	-
Female		-9,988.11***	-10,294.01**	-5,684.56
		(2,719.51)	(3,199.59)	(3,087.37)
Married		11,265.08***	9,702.48**	-942.61
		(3,130.32)	(3,670.22)	(3,589.15)
Household size		946.80	361.68	-2,767.80*
		(1,174.68)	(1,391.18)	(1,349.08)
Father’s education			838.34	430.49
			(877.63)	(857.02)
Mother’s education			1,204.84	1,439.29
			(901.08)	(878.49)
Family income at age 10			6,436.41***	2,719.27*
			(1,426.19)	(1,384.21)
Household income (Per £1000)				879.71***
				(42.66)
Constant	30,289.40***	25,550.43***	-28,681.21	-32,924.53*
	(1,709.25)	(4,049.99)	(15,135.49)	(14,748.31)
Observations	5,341	5,341	4,072	3,894
*R* ^*2*^	.018	.025	.034	.128

Standard errors in parentheses

*** *p* < .001,

** *p* < .01,

* *p* < .05

The results without covariates are visualized in Fig 6. We find that the relationship between cognitive ability and savings displays a trend of exponential growth, indicating that greater levels of cognitive ability are associated with even greater accumulation of savings. However, this non-linear effect is no longer significant after controlling for income.

#### Asset-to-income ratio

We next assess the functional form of the relationship between cognitive ability and a household’s asset-to-income ratio as an additional indicator of wealth accumulation. We find that the cubic term of cognitive ability does not significantly predict the asset-to-income ratio (*b*^*3*^ = 0.03, *se* = 0.04, *t*(5,059) = 0.84, *p* = .399). We therefore tested the squared term in the reported models (Table 7). Following our approach used for the debt-to-income ratio, we do not report a model that controls for household income, as this variable is a component of the dependent variable. Consistent with the findings for total savings, we find that the relationship between cognitive ability and the asset-to-income ratio follows a quadratic polynomial form (Fig 7), with the squared term Cognitive Ability^2^ significantly predicting the asset-to-income ratio in the first two models (e.g., Model 1—without controls, *b*^*2*^ = 0.16, *se* = 0.07, *t*(5,060) = 2.35, *p* = .019). However, the squared term is no longer significant at the 5% level when controlling for adult covariates and childhood socioeconomic status (Model 3 –adult and childhood controls, *b*^*2*^ = 0.16, *se* = 0.09, *t*(3,860) = 1.92, *p* = .055). The findings suggest that the quadratic term of cognitive ability may have a curvilinear relationship with the asset-to-income ratio, but the effect is dependent on other factors.

**Table 7:** OLS regression models for the relationship between cognitive ability and the asset-to-income ratio.

VARIABLES	(1)	(2)	(3)
	Asset-to-income ratio (Age 46)	Asset-to-income ratio (Age 46)	Asset-to-income ratio (Age 46)
Cognitive ability	0.52***	0.53***	0.50***
	-0.09	(0.091)	(0.12)
Cognitive ability^2^	0.16*	0.15*	0.16
	-0.07	(0.067)	(0.09)
Cognitive ability^3^	-	-	-
Female		-0.40*	-0.44*
		(0.17)	(0.20)
Married		0.05	0.08
		(0.20)	(0.23)
Household size		-0.14	-0.25**
		(0.07)	(0.09)
Father’s education			0.11
			(0.06)
Mother’s education			0.03
			(0.06)
Family income at age 10			0.16
			(0.09)
Household income (Per £1000)			
Constant	1.30***	1.93***	-0.57
	-0.11	(0.25)	(0.97)
Observations	5,063	5,063	3,869
*R* ^*2*^	.008	.010	.017

Standard errors in parentheses

*** *p* < .001,

** *p* < .01,

* *p* < .05

#### Pension scheme membership

Next, we assess to what extent cognitive ability predicts whether an individual is a member of a pension scheme. Data on pension scheme membership were only gathered in the 2012 wave of the British Cohort Study, so the results reported indicate pension scheme membership at age 42. We present these analyses in Table 8.

**Table 8:** OLS logistic regression models for the relationship between cognitive ability and pension scheme ownership.

VARIABLES	(1)	(2)	(3)	(4)
	Has pension scheme (Age 42)	Has pension scheme (Age 42)	Has pension scheme (Age 42)	Has pension scheme (Age 42)
Cognitive ability	0.69***	0.65***	0.52***	0.36***
	(0.05)	(0.05)	(0.06)	(0.06)
Cognitive ability^2^	-0.01	-0.02	-0.00	-0.00
	(0.03)	(0.03)	(0.03)	(0.04)
Cognitive ability^3^	-0.04**	-0.04**	-0.02	-0.01
	(0.01)	(0.01)	(0.02)	(0.02)
Female		-0.35***	-0.34***	-0.16*
		(0.06)	(0.07)	(0.07)
Married		0.62***	0.58***	0.28***
		(0.06)	(0.08)	(0.08)
Household size		-0.07**	-0.07**	-0.06*
		(0.02)	(0.03)	(0.03)
Father’s education			0.05	0.05
			(0.02)	(0.02)
Mother’s education			0.00	-0.01
			(0.02)	(0.03)
Family income at age 10			0.18***	0.14***
			(0.03)	(0.03)
Household income (Per £1000)				0.04***
				(0.00)
Constant	1.20***	1.26***	-0.09	-0.55
	(0.04)	(0.09)	(0.41)	(0.43)
Observations	7,145	7,145	5,363	5,363
Pseudo *R*^*2*^	.052	.069	.069	.144

Standard errors in parentheses

*** *p* <. 001,

** *p* <. 01,

* *p* < .05

We find that for the first three model specifications, the relationship between cognitive ability and pension scheme membership follows a cubic polynomial form, with the cubic term Cognitive Ability^3^ significantly predicting total debt in these models (e.g., Model 1 –without controls, *b*^*3*^ = -0.04, *se* = 0.01, *z* = -2.83, *p* = .005). However, the cubic term of cognitive ability is no longer significant once we control for childhood socioeconomic status and household income (Model 4 –all controls, *b*^*3*^ = -0.03, *se* = 0.02, *z* = -1.52, *p* = .128). We do still find a significant positive association between cognitive ability and pension scheme membership, beyond the effect of income.

The results are visualized in Fig 8. We find that the relationship between cognitive ability and membership of a pension scheme is characterized by a strong positive relationship (above ~ -2 SD), followed by diminishing returns, where the effect levels off at high levels of cognitive ability. However, this non-linear effect is no longer significant after controlling for income.

#### Holds investment account

The relationship between cognitive ability and holding an investment account shows a similar pattern as that between cognitive ability and being a member of a pension scheme (Table 9). Whereas we find initial evidence of a non-linear relationship between cognitive ability and holding an investment account (Model 1—without controls, *b*^*3*^ = -0.03, *se* = 0.01, *z* = -2.06, *p* = .039), the cubic term of cognitive ability is no longer significant when controlling for income (Model 4 –all controls, *b*^*3*^ = -0.03, *se* = 0.02, *z* = -1.42, *p* = .155). However, we do find that individuals with greater cognitive ability remain more likely to hold an investment account (Model 4 –all controls, *b* = 0.42, *se* = 0.06, *z* = 6.48, *p* < .001).

**Table 9:** OLS logistic regression models for the relationship between cognitive ability on holding an investment.

VARIABLES	(1)	(2)	(3)	(4)
	Investment account (Age 46)	Investment account (Age 46)	Investment account (Age 46)	Investment account (Age 46)
Cognitive ability	0.63***	0.60***	0.44***	0.42***
	(0.05)	(0.05)	(0.06)	(0.06)
Cognitive ability^2^	0.02	0.02	-0.01	-0.02
	(0.03)	(0.03)	(0.03)	(0.03)
Cognitive ability^3^	-0.03*	-0.03*	-0.02	-0.03
	(0.01)	(0.01)	(0.02)	(0.019)
Female		-0.24***	-0.24***	-0.25***
		(0.06)	(0.07)	(0.07)
Married		0.69***	0.69***	0.53***
		(0.07)	(0.08)	(0.08)
Household size		0.03	0.01	-0.02
		(0.03)	(0.03)	(0.03)
Father’s education			0.01	0.01
			(0.02)	(0.02)
Mother’s education			0.03	0.04
			(0.02)	(0.02)
Family income at age 10			0.19***	0.13***
			(0.03)	(0.03)
Household income (Per £1000)				0.01***
				(0.00)
Constant	-1.08***	-1.50***	-2.74***	-2.91***
	(0.04)	(0.09)	(0.30)	(0.32)
Observations	6,328	6,328	4,796	4,450
Pseudo *R*^*2*^	.043	.063	.065	.090

Standard errors in parentheses

*** *p* < .001,

** *p* < .01,

* *p* < .05

**Fig 9:**
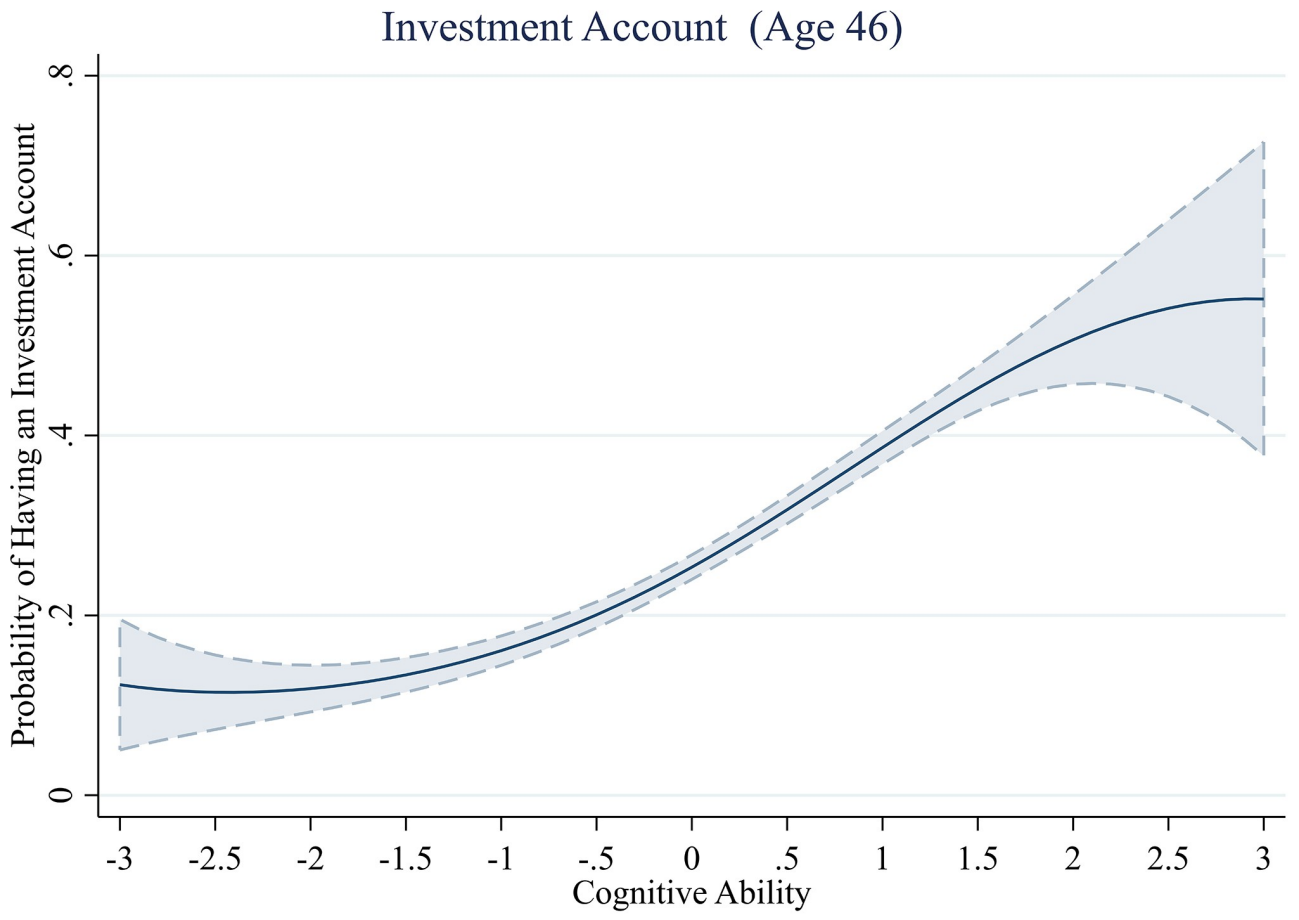
Probability of holding an investment account across levels of cognitive ability.

The results are visualized in Fig 9. We find that the relationship between cognitive ability and holding an investment account is characterized by a strong positive relationship (above ~ -1 SD), followed by diminishing returns at the highest levels of cognitive ability (above ~ 1.5 SD). However, this non-linear effect is no longer significant after controlling for childhood SES or income.

### Subjective financial well-being

**Fig 10:**
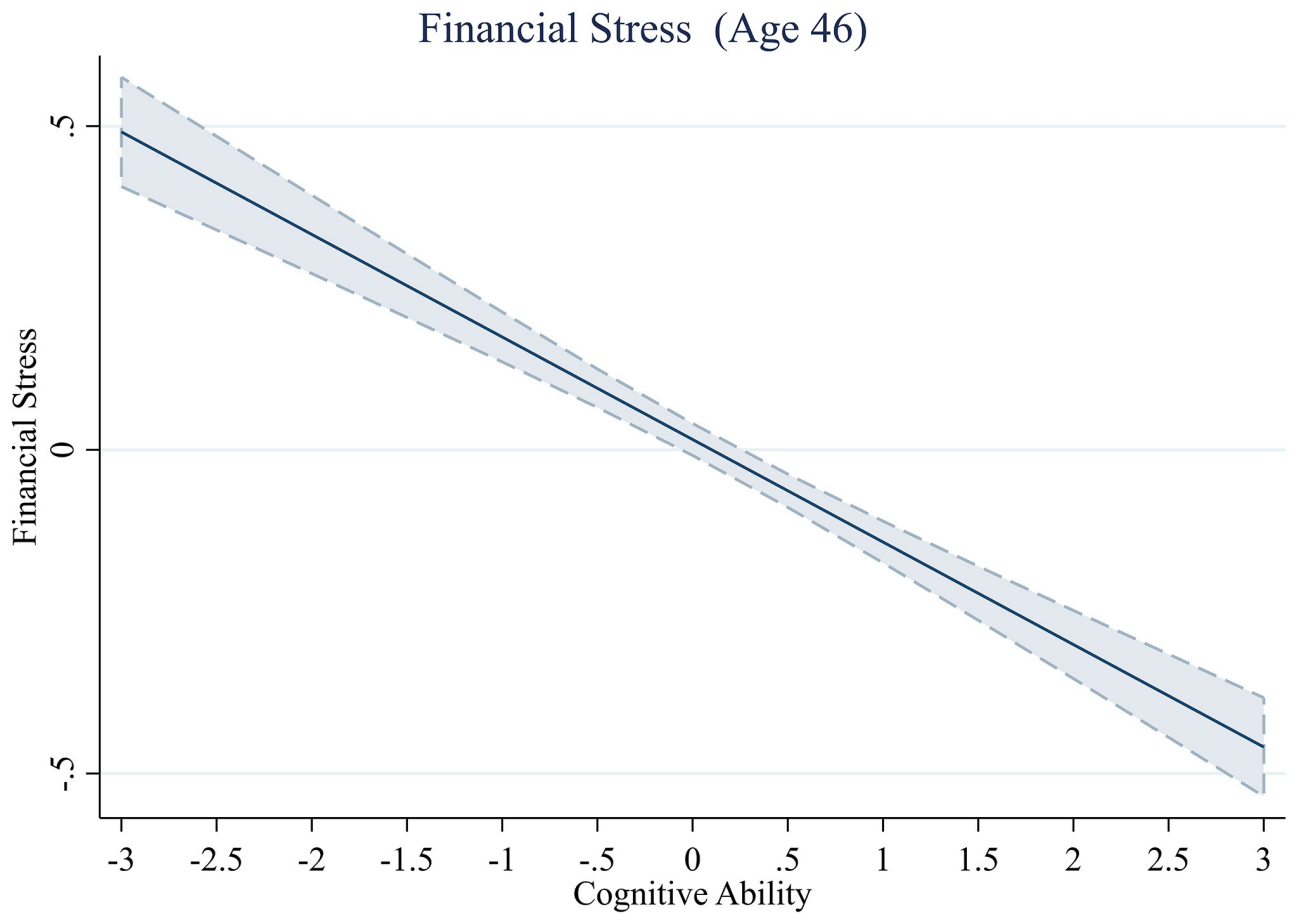
Financial stress across levels of cognitive ability.

Next, we describe the results for individual’s subjective assessments of their financial well-being. We do not find evidence of non-linear relationships between cognitive ability and financial distress (Table 10, Fig 10). However, we do find that those higher in cognitive ability tend to worry less about money than those lower in cognitive ability–even after controlling for income. In Model 4, each additional SD unit increase in cognitive ability is associated with a -0.06 SD reduction in financial stress (*b* = -0.06, *se* = .02, *t*(4,441) = -4.05, *p* < .001).

**Table 10:** OLS regression models for the relationship between cognitive ability and financial stress.

VARIABLES	(1)	(2)	(3)	(4)
	Financial stress (Age 46)	Financial stress (Age 46)	Financial stress (Age 46)	Financial stress (Age 46)
Cognitive ability	-0.16***	-0.14***	-0.10***	-0.06***
	(0.01)	(0.01)	(0.02)	(0.02)
Cognitive ability^2^	-	-	-	-
Cognitive ability^3^	-	-	-	-
Female		0.01	0.01	0.00
		(0.02)	(0.03)	(0.03)
Married		-0.40***	-0.35***	-0.25***
		(0.03)	(0.03)	(0.032)
Household size		0.02	0.03*	0.05***
		(0.01)	(0.01)	(0.01)
Father’s education			-0.01	-0.01
			(0.01)	(0.01)
Mother’s education			0.02*	0.02*
			(0.01)	(0.01)
Family income at age 10			-0.07***	-0.04**
			(0.01)	(0.01)
Household income (Per £1000)				-0.01***
				(0.00)
Constant	0.02	0.19***	0.31*	0.30*
	(0.01)	(0.04)	(0.13)	(0.13)
Observations	6,321	6,321	4,791	4,450
*R* ^*2*^	.023	.057	.054	.124

Standard errors in parentheses

*** *p* < .001,

** *p* < .01,

* *p* < .05

## General discussion

This study aimed to examine the shape of the relationship between childhood cognitive ability and various indicators of financial well-being later in life. In contrast to previous studies, we explicitly tested the functional form of these relationships and found that it varies across different indicators of financial well-being. Most notably, we found an inverse U-shaped relationship between cognitive ability and debt, with those who are both low and high in cognitive ability having the lowest total debt and probability of holding revolving credit card debts, and those with average levels of cognitive ability having the highest debt. We also found an exponential relationship with the amount of money participants saved, but this effect is no longer significant when we control for the effect of income. The non-linear relationship between cognitive ability and the indicators of wealth was mostly seen at the extreme ends of the cognitive ability distribution, whereas a linear trend was seen for most people. Similarly, despite our efforts to test for potential non-linearities, the data revealed that a linear trend provides the most accurate description of the relationship between cognitive ability and financial stress.

The inverse U-shaped relationship between cognitive ability and debt accumulation that we report may reconcile seemingly inconsistent prior findings on the association between cognitive ability and debt. On the one hand, there is evidence that individuals with higher cognitive ability are more debt-averse [27], while on the other, research has found that those with high cognitive ability are more likely to hold debt [3]. By moving beyond the assumption that the association between cognitive ability and debt is linear, these seemingly contradictory findings can be better understood. While cognitive ability may be associated with greater debt aversion, individuals with low cognitive ability may lack access to credit [68]. This would result in limited debt accumulation by those with low cognitive ability, even if they would prefer greater credit access. Whereas we may have expected those with higher cognitive ability to be more likely to hold debt as they more easily navigate and understand complex financial products [3], it may instead be the case that at the highest levels of cognitive ability, debt accumulation is lower because individuals have less desire to take out credit.

Our findings on the relationship between cognitive ability and debt accumulation differ from previous research, which has reported that individuals with lower cognitive ability are more likely to hold credit card debt [28]. This discrepancy may be explained by the fact that we measure general cognitive ability, while the other study [28] uses a measure of executive functioning, a specific form of cognitive ability. However, our results show similar patterns to those found by previous research [9] that used data from the National Longitudinal Survey of Youth (NLSY79) to test for non-linear relationships between cognitive ability and negative financial behaviors, such as being late paying bills and going bankrupt. In this study [9] the highest rates of these behaviors were found to occur in the middle of the cognitive ability distribution, which aligns with our findings on debt amount and revolving credit card debt. However, this pattern is not reflected in our measures of savings or subjective financial well-being.

Our findings demonstrate a positive correlation between cognitive ability and financial well-being, as measured by various indicators of wealth accumulation. High cognitive ability individuals generally have higher savings balances, a larger asset-to-income ratio, a greater likelihood of being enrolled in a pension plan, and are more likely to hold an investment account. This may reflect their ability to navigate and understand complex financial products. However, it is important to note that the relationship between cognitive ability and wealth accumulation may be confounded by income. Our analyses suggest that income plays a potentially influential role in this relationship, as the inclusion of income as a covariate reduces the strength of the effect of cognitive ability on savings balances and pension scheme membership. Furthermore, after including income, the initial non-linear relationships found (e.g. exponential for total savings and asset-to-income ratio, diminishing returns for pension scheme membership) became non-significant. Controlling for income allowed us to isolate the direct relationship between cognitive ability and savings, and we find that this relationship is best described by a linear trend.

Our study also finds that individuals with greater cognitive ability report lower levels of financial stress. We observed a linear trend in this relationship, indicating that, even for individuals with high cognitive ability, an increase in their cognitive ability scores would be associated with a decrease in financial stress. However, it is important to acknowledge that financial stress is only one aspect of financial well-being, and incorporating objective indicators is crucial to obtaining a comprehensive understanding. Previous literature has shown that individuals’ subjective assessment of their financial situation may not always align with their actual financial status [69]. Simply lacking financial stress does not necessarily indicate financial well-being, as it could result from avoidance of financial responsibilities [70]. Overall, we contribute to the rich literature on subjective financial well-being [e.g., 16, 63] by demonstrating that cognitive ability predicts not only objective measures but also *subjective* assessments of one’s financial situation. This is especially important given the detrimental consequences of financial stress on health and overall well-being [71]. Our study highlights the importance of cognitive ability as a potential protective factor against financial stress and underscores the need for a holistic approach to understanding financial well-being. Our research shows that cognitive ability plays a distinct role in financial well-being beyond the influence of income. We provide evidence of this by illustrating that cognitive ability has unique explanatory power in predicting financial outcomes, even when controlling for income. In the online appendix (Web Appendix B in S1 File), we provide supplementary analyses showing that cognitive ability is positively related to income, consistent with previous research [e.g., 15].

Specifically, our findings reveal a non-linear relationship between cognitive ability and income, with diminishing returns at lower levels of cognitive ability and an exponential trend at higher levels. While higher levels of income are likely one route through which cognitive ability leads to increased financial well-being, these findings underscore the importance of considering cognitive ability as a separate predictor of financial well-being, rather than solely relying on income as an explanation.

Finally, our study demonstrates that, in addition to cognitive ability and income, family background plays a crucial role in shaping an individual’s financial outcomes. We observe that family income at age 10 is a significant predictor of savings, pension plan membership, investment account holding, and financial stress at age 46. Our findings are consistent with previous research on the intergenerational transmission of wealth [e.g., 72], which describes how financial well-being is passed down from one generation to the next, as well as research on financial socialization [73]. These results emphasize the importance of considering the impact of family background on financial well-being, in addition to cognitive ability and income, when designing interventions or policies aimed at improving financial outcomes.

### Theoretical and policy implications

Theoretically, our results suggest that cognitive ability plays an important role in financial decision-making, enabling individuals to navigate and understand complex financial products. As the complexity of financial products that consumers are exposed to increases [3], cognitive ability is generally a valuable resource that can enable positive financial decision-making. Additionally, our findings imply that the relationship between cognitive ability and financial well-being may depend on access to financial products and opportunities. We emphasize that our results do not imply that individuals with lower cognitive abilities cannot achieve financial well-being.

Our research contributes to prior research on the association between cognitive ability and financial well-being in several ways. First, we study possible non-linear relationships between cognitive ability and financial well-being. Our work expands the robustness of prior work [9] by studying non-linear effects for a broader range of indicators of financial well-being, including subjective perceptions of financial well-being in another cohort and another country. Second, we provide a novel perspective on the association between cognitive ability and financial outcomes by controlling for the effect of income. Our work demonstrates that cognitive ability is associated with financial outcomes when controlling for the well-documented effect of cognitive ability on income [15]. A specific strength of our paper is that we use a high-quality measure of cognitive ability to estimate the impact of cognitive ability more precisely in childhood on adult financial outcomes. Previous research has often used proxies of cognitive ability, such as the cognitive reflection test [25, 27, 40]. Using a high-quality measure of cognitive ability, measured before school streaming occurred [45], allows us to estimate the impact of cognitive ability more precisely in childhood on adult financial outcomes. Whereas prior research has measured cognitive ability in adulthood or uses proxy measures of cognitive ability, we demonstrate how a measure of cognitive ability in childhood, which is not confounded by financial experience, is associated with financial outcomes in adulthood.

Our study reveals that cognitive ability is a reliable predictor of financial well-being; however, we acknowledge that other unmeasured characteristics may also influence financial outcomes. The modest R^2^ values of our regression models suggest that there are other significant factors that we did not account for. To identify such factors, future studies may use a data-driven approach like least absolute shrinkage and selection operator (LASSO) analysis, a method that performs variable selection to enhance the prediction accuracy and interpretability of the resulting model [74]. One such unmeasured factor that may influence financial well-being is financial experience, which has been shown to compensate for cognitive decline due to aging [75]. Another factor is financial literacy, which is known to predict financial well-being and reduce financial errors [25, 38]. Measuring financial literacy separately may explain financial decision-making and financial outcomes beyond the general measure of cognitive ability used in this study. Thus, understanding the interplay between cognitive ability, financial experience, and financial literacy is crucial in promoting sound financial decision-making and, ultimately, achieving financial well-being.

Our findings on the relationship between cognitive ability and financial well-being have significant policy implications. By identifying the functional form of these relationships, we gain insights into which individuals may benefit the most from interventions aimed at improving financial well-being. For instance, our results suggest that policies aimed at limiting overexposure to credit should focus on individuals with average cognitive abilities. These individuals are more likely to have access to debt than those with the lowest cognitive abilities, and they may have a greater need to access debt than those with the highest cognitive abilities. This highlights the importance of targeting interventions to specific subgroups of the population rather than applying a one-size-fits-all approach [76].

The positive effect of cognitive ability on financial outcomes highlights the need for interventions to address the financial literacy and decision-making skills of individuals with lower cognitive abilities. Such interventions may include financial education programs as well as policies that increase access to financial products and opportunities for those with lower cognitive ability. Additionally, our findings call for further research to better understand the non-linear relationship between cognitive ability and financial well-being in order to inform the design of more effective policies and interventions.

While our research does not investigate the specific mechanisms underlying the non-linear relationship between cognitive ability and financial well-being, gaining such insight is essential for informing interventions and applying our findings to improve financial outcomes. The causal mechanisms through which cognitive ability impacts financial decision-making are complex and multifaceted. Cognitive ability and the time preferences associated with cognitive ability are closely linked to educational and occupational attainment [23], which in turn can influence the accumulation of wealth [as reviewed by 77]. Furthermore, cognitive ability may affect an individual’s access to financial products via multiple routes. First, through its effect on income, cognitive ability may increase an individual’s eligibility for specific financial products, such as loans. Second, the cost of information acquisition is lower for individuals with higher cognitive abilities, which lowers the cost of taking on novel financial products, such as stocks. Individuals who have more access to financial products such as investments have a stronger incentive to gain knowledge about these topics, which in turn may increase their financial literacy [25]. While access to financial resources can be a positive factor, it may also have negative consequences if it leads to overexposure to credit or exposure to detrimental financial products [68]. Some authors [78] argue that individuals at lower levels of financial literacy may be aware of the insufficiency of their financial knowledge, and therefore attempt to avoid financial mistakes. Through this process, cognitive ability can have a positive effect on financial well-being, as it reduces the barriers for the take-up of wealth-building products, while simultaneously, the lower barrier may simultaneously explain higher levels of debt through an increased take-up of bad sources of debt at average levels of cognitive ability. Further research that includes measures to isolate these individual components can help elucidate these complex mechanisms.

Our research also highlights the importance of considering multiple indicators of financial well-being when examining the relationship between cognitive ability and financial outcomes. While traditional measures, such as debt accumulation, may suggest that individuals with lower cognitive abilities have more favorable financial circumstances, our findings on indicators such as savings and subjective financial well-being suggest otherwise. This highlights the importance of considering both objective and subjective measures of financial well-being to fully understand the complex relationship between cognitive ability and financial outcomes. As subjective financial well-being is strongly associated with overall well-being [63, 79], the role of subjective perceptions of financial well-being should not be underestimated. Additionally, our findings suggest that different interventions may be necessary for individuals at different levels of cognitive ability to improve financial well-being. Therefore, it is crucial for policymakers to consider the functional form of the relationship between cognitive ability and financial well-being when designing interventions to improve financial outcomes for all individuals.

In practice, it may not be feasible to individually assess the cognitive ability of all members of society to determine which individuals would benefit most from a policy intervention. However, this challenge can be addressed by utilizing proxies for cognitive ability, such as education level [80], or by utilizing digital footprints, such as online behavior or transaction histories, to predict cognitive ability at scale [81–83].

### Limitations and future research

Some limitations should be kept in mind when interpreting the current findings. Due to the nature of the dataset, we only have information on the cognitive ability of one individual in the household. Whereas we find positive associations between cognitive ability and individual financial outcomes, such as whether an individual is a member of a pension plan, some of the financial well-being measures, such as income, wealth and debt, are measured at the household level. However, we do not believe this invalidates our results, as there is generally a strong correlation between the cognitive ability of partners [84].

To gain a more complete understanding of the factors contributing to the inverted U-shaped relationship between cognitive ability and debt, it is important for future research to utilize a more nuanced approach to measuring debt. While our study focused on revolving debts and excluded mortgage debts, with the aim of identifying the debt associated with suboptimal financial decision-making, debt may not always signify poor financial decision-making. In Web Appendix C in S1 File, we report additional analyses that demonstrate that in our study, debt is associated with financial stress and that the inverse U-shape relationship between cognitive ability is robust when excluding student debt, which can be seen as a positive use of debt. Therefore, it is essential to consider the type and purpose of debt, as well as an individual’s overall financial resources. Although our study considered the debt-to-income ratio, more detailed measures of debt, such as those used in the Household, Income, and Labor Dynamics in Australia (HILDA) survey [85], which includes information about credit card repayment frequency, could offer more insight into the relationship between debt and financial well-being. Such an analysis would provide a more nuanced understanding of how the purpose and frequency of debt repayment can affect an individual’s financial well-being.

In interpreting our findings, it is also important to consider the limitations of our measurements. The continuous indicators of financial well-being that we measured, such as debt, debt-to-income ratio, savings, and asset-to-income ratio, have a non-Gaussian distribution, which can lead to outliers that may bias our analyses. To address this issue, we used a winsorizing approach that reduces the impact of outliers (i.e., the top 1%) on the results while preserving the distribution of the data. However, this approach assumes that extreme values are indeed outliers, which is a matter of contention [86]. Future research using registration data instead of self-reported financial data could provide a more accurate measure of financial outcomes and shed light on whether the functional forms of the relationship between cognitive ability and financial outcomes hold when measurement error is minimized.

A further limitation is that the extent to which our findings are generalizable to populations beyond the British sample studied is unclear. In particular, cross-country variations in the level of state intervention in the financial lives of citizens, such as through the generosity of welfare systems and the degree of regulation of the financial system, are likely to impact the patterns of associations found between cognitive abilities and financial well-being. For example, in the United States, welfare is less generous and conditional on employment to a greater extent than in Europe [87]. We study the association between cognitive abilities and financial well-being in the United Kingdom, a European-style welfare system. A high level of state intervention may reduce the strength of the relationship, as policies such as mandatory auto-enrolment in retirement plans (e.g., the 2008 Pensions Act) may reduce inequalities across groups. Thus, the strength and direction of the association between cognitive ability and financial well-being may be contingent on the welfare system of a given country.

The present study does not explore the impact of personality on financial outcomes, despite a broad literature demonstrating the important role of personality in shaping both consumption [88–94] and personal finance behaviors [76, 95–99]. Practically, limitations in the available data limited our ability to include measures of personality in the analyses. Personality has been measured in childhood but just as a proxy from other measures. Theoretically, our study was driven by the belief that cognitive ability plays a more proximal role in financial decision-making and outcomes, and it is challenging to identify which specific personality traits to measure and how to interpret the data meaningfully. However, research has shown that personality traits have unique explanatory power beyond the role of intelligence. For example, in a high-IQ sample, conscientiousness and extraversion positively predict men’s lifetime earnings, whereas agreeableness and earnings are negatively related [100]. Trait conscientiousness is also a determinant of wealth [19, 21], controlling for differences in cognitive ability. Although there is academic debate about the relative importance of cognitive ability and personality in predicting life outcomes [see 101, 102], little is known about how cognitive and non-cognitive factors interact to achieve financial well-being. Future research could explore how specific personality traits, such as conscientiousness, extraversion, and agreeableness, enhance or attenuate the positive effect of cognitive ability on financial well-being.

## Conclusion

In conclusion, our study provides new insights into the relationship between childhood cognitive ability, financial resources, and financial outcomes in adulthood. Our findings suggest that childhood cognitive ability is a significant predictor of financial outcomes in adulthood, and that this relationship is not solely driven by differences in income. We further find that these relationships are not consistently characterized by linearity, with debt characterized by an inverse U-shape. The functional form of the relationship between cognitive ability and financial well-being depends on the type of outcome studied. These results have important implications for policymakers and financial advisors who seek to improve financial well-being and reduce financial inequality. By understanding the complex interplay between cognitive ability, financial resources and financial outcomes, targeted interventions can be developed to help those who are at the greatest risk of experiencing financial hardship. Our study also highlights the need for further research in this area, including exploring the underlying mechanisms that explain the relationship between cognitive ability and financial outcomes and examining the role of other factors such as financial literacy and risk attitudes. By doing so, we can obtain a more complete understanding of the links between these variables and advance our understanding of how to enhance financial well-being across the lifespan.

## Supporting information

S1 File (DOCX)
