# Supplementary material for: Understanding the functional form of the relationship between childhood cognitive ability and adult financial well-being
Source: PLoS One. 2023 Jun 7;18(6):e0285199. doi: 10.1371/journal.pone.0285199 (PMC10246798; doi:10.1371/journal.pone.0285199)

**Web Appendix A – Results for 2012 Wave**

Our main analyses focus on the most recent wave of the British Cohort Study, which was conducted in 2016. To demonstrate the robustness of our results, we report similar analyses for the 2012 below. We do not report findings for having a retirement plan, as this data is only available for the 2012 wave.

As demonstrated in Figure A1, we find similar trends for the association between cognitive ability and adult financial well-being that are robust over time across all financial outcomes measured. Whereas the functional form of the association between cognitive ability and the debt-to-income ratio was difficult to derive from the analyses for the 2016 wave due to the large confidence intervals at extreme ends of the cognitive ability distribution, the analyses from 2012 replicate the inverse U-shape that we find across all other debt measurements. The only notable difference in patterns across the waves can be found in financial stress. Whereas this pattern was negatively linear in 2016, we find non-linear effects in 2012 (Table A1, Column 5). However, after adding adult demographic characteristics, childhood socioeconomic status, and income, the cubic effect becomes non-significant. Therefore, our conclusions, based on the analyses of the 2016 wave, remain robust.

**Figure A1.** Comparison of financial outcomes over levels of cognitive ability for 2012 (Age 42) and 2016 (Age 46).

| Total debt | |
| --- | --- |
|  | 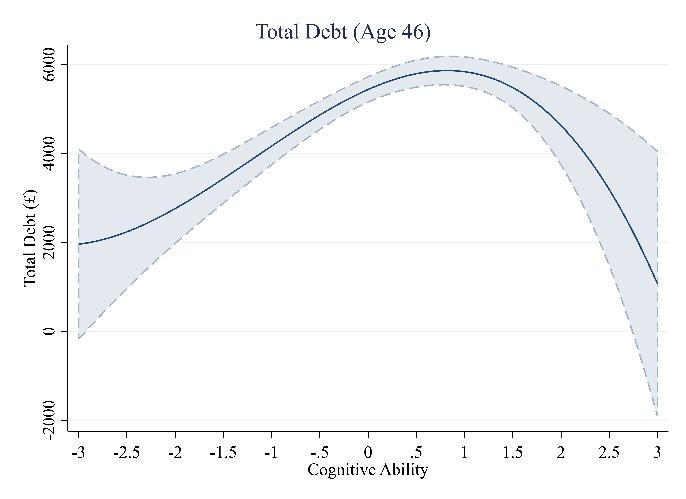 |
| Debt-to-income ratio | |
| 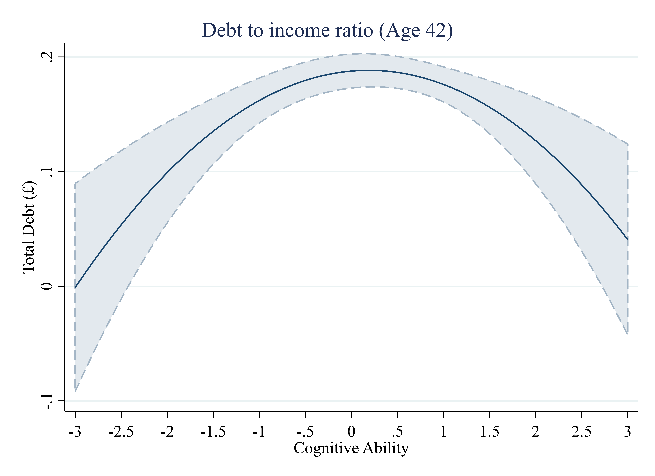 | 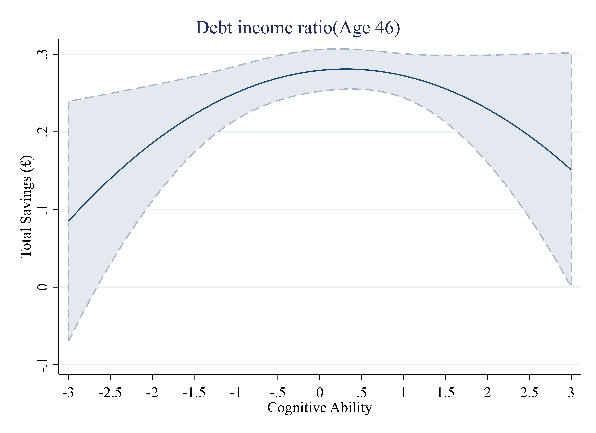 |
| Credit card / store card debt | |
|  | 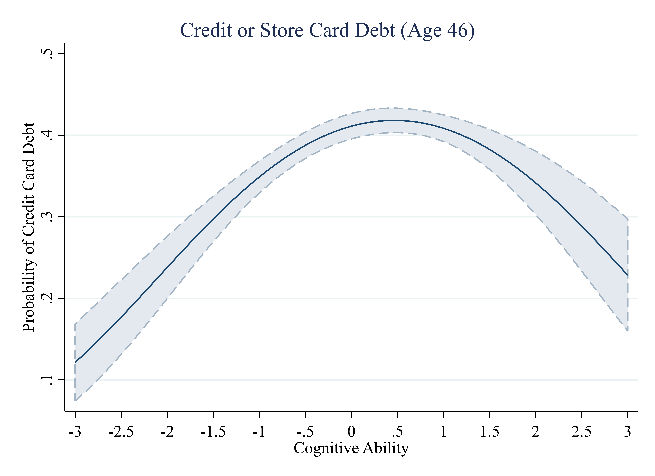 |
| Total savings | |
|  | 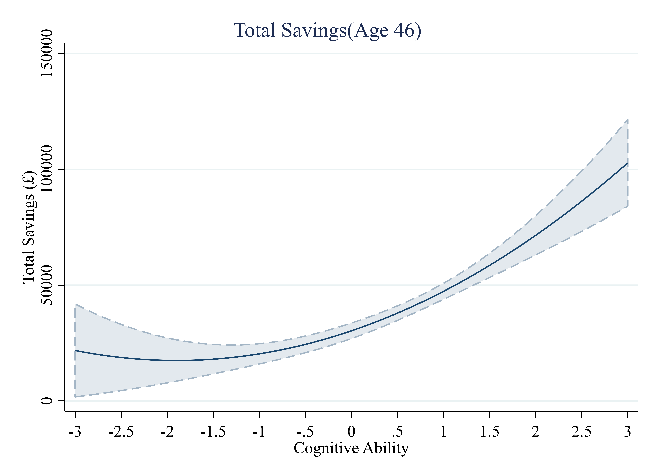 |
| Asset-to-income ratio | |
| 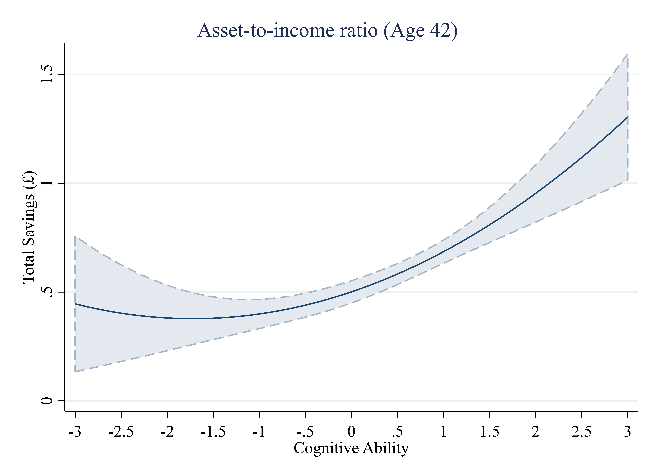 | 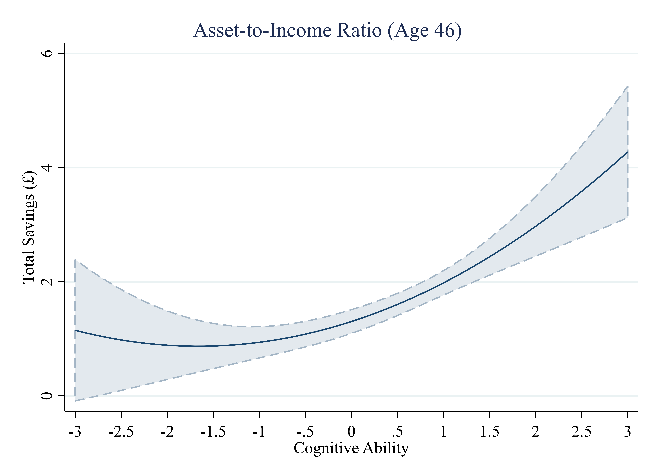 |
| Investment account | |
|  | 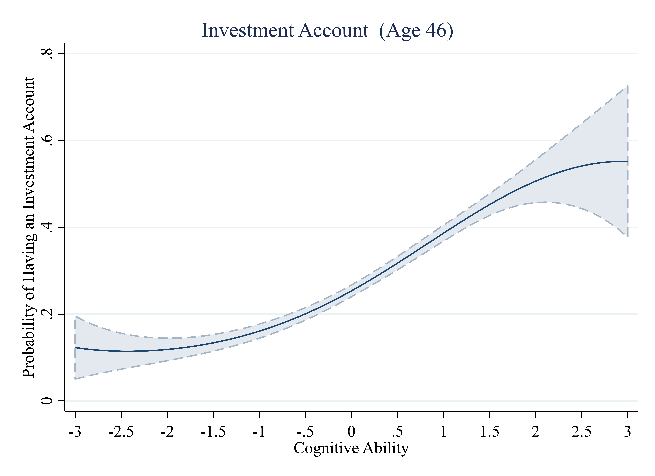 |
| Financial stress | |
|  | 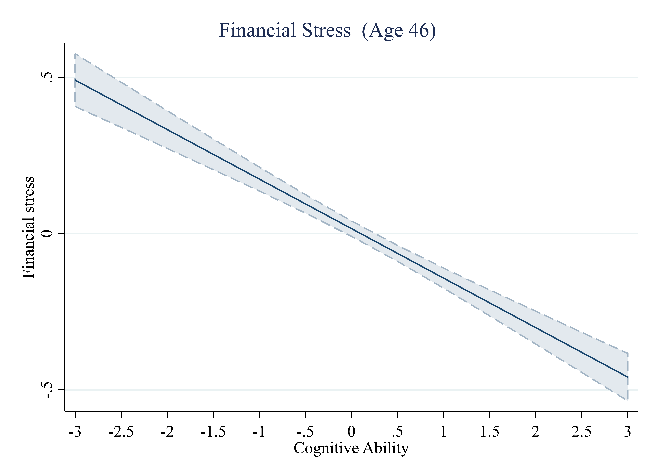 |
| Income | |
|  | 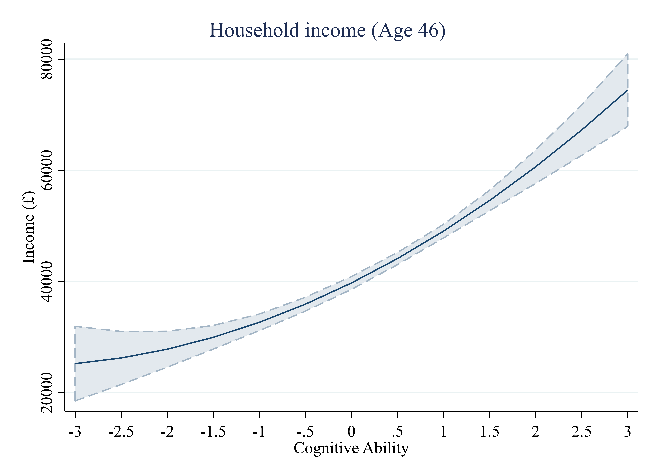 |

**Table A1.** *OLS regression and logistic models for the relationship between cognitive ability and financial outcomes at age 42 without controls*

|  | (1) | (2) | (3) | (4) | (5) | (6) | (7) | (8) |
| --- | --- | --- | --- | --- | --- | --- | --- | --- |
| VARIABLES | Total  debt | Debt-to-income ratio | Credit card debt | Total  savings | Asset-to-income ratio | Investment account | Financial stress | Income |
|  |  |  |  |  |  |  |  |  |
| Cognitive ability | 983.87*** | 0.01 | 0.09*** | 9,046.69*** | 0.14*** | 0.75*** | -0.20*** | 8,476.88*** |
|  | (186.95) | (0.01) | (0.03) | (640.23) | (0.02) | (0.04) | (0.02) | (379.31) |
| Cognitive ability^2^ | -404.79*** | -0.02*** | -0.11*** | 2,782.21*** | 0.04* | -0.02 | -0.01 | 755.81*** |
|  | (95.68) | (0.01) | (0.02) | (475.94) | (0.02) | (0.02) | (0.01) | (213.42) |
| Cognitive ability^3^ | -132.26* | - | - | - | - | -0.04*** | 0.01* | -229.08* |
|  | (51.71) | - |  |  | - | (0.01) | (0.01) | (102.25) |
|  |  |  |  |  |  |  |  |  |
| Constant | 4,824.13*** | 0.19*** | -0.36*** | 15,333.62*** | 0.50*** | -1.62*** | 0.03* | 19,005.09*** |
|  | (144.95) | (0.01) | (0.03) | (763.42) | (0.03) | (0.03) | (0.01) | (321.90) |
|  |  |  |  |  |  |  |  |  |
| Observations | 5,687 | 4,368 | 7,240 | 5,966 | 4,931 | 11,611 | 7,229 | 11,611 |
| *R²* | .008 | .004 | .004 | .037 | .010 | .056 | .027 | .074 |
| Standard errors in parentheses | | | | | | | | |
| *** *p* < .001, ** *p* < .01, * *p* < .05 | | | | | | | | |

**Table A2.** *OLS regression and logistic models for the relationship between cognitive ability and financial outcomes with controls*

|  | (1) | (2) | (3) | (4) | (5) | (6) | (7) | (8) | |
| --- | --- | --- | --- | --- | --- | --- | --- | --- | --- |
| VARIABLES | Total debt | Debt-to-income ratio | Credit card debt | Total savings | Asset-to-income ratio | Investment account | Financial stress | Income | |
|  |  |  |  |  |  |  |  |  | |
| Cognitive ability | 800.19*** | 0.02 | 0.08* | 4,223.16*** | 0.10*** | 0.41*** | -0.09*** | 7,783.91*** | |
|  | (236.31) | (0.01) | (0.03) | (803.26) | (0.028) | (0.06) | (0.02) | (654.44) | |
| Cognitive ability^2^ | -453.76*** | -0.02** | -0.09*** | 1,607.99** | 0.02 | 0.01 | -0.00 | 510.00 | |
|  | (116.89) | (0.01) | (0.03) | (567.24) | (0.02) | (0.03) | (0.01) | (329.80) | |
| Cognitive ability^3^ | -112.37 |  | - | - |  | -0.03 | 0.01 | -461.53* | |
|  | (65.96) |  |  |  |  | (0.02) | (0.01) | (185.77) | |
| Female | -928.73*** | -0.01 | -0.09 | -3,360.40* | -0.04 | -0.09 | -0.08** | -8,091.86*** | |
|  | (276.88) | (0.01) | (0.06) | (1,418.74) | (0.05) | (0.06) | (0.03) | (772.76) | |
| Married | 930.95** | -0.01 | 0.12 | 2,239.00 | 0.11 | 0.35*** | -0.22*** | 12,012.47*** | |
|  | (318.90) | (0.02) | (0.07) | (1,630.65) | (0.06) | (0.08) | (0.03) | (879.53) | |
| Household size | 332.03** | 0.02** | 0.04 | -733.39 | -0.01 | -0.00 | 0.05*** | -125.64 | |
|  | (113.67) | (0.01) | (0.02) | (574.23) | (0.02) | (0.03) | (0.01) | (317.14) | |
| Father’s education | -5.43 | -0.01 | -0.03 | 985.87* | 0.03* | 0.03* | -0.00 | 199.22 | |
|  | (71.10) | (0.00) | (0.02) | (386.30) | (0.01) | (0.02) | (0.01) | (206.88) | |
| Mother’s education | 66.51 | 0.01 | -0.00 | 354.28 | -0.01 | -0.00 | 0.00 | 504.07* | |
|  | (78.30) | (0.00) | (0.02) | (401.56) | (0.01) | (0.02) | (0.01) | (229.22) | |
| Family income at age 10 | -135.93 | -0.02** | -0.03 | 2,188.70*** | 0.04 | 0.17*** | -0.04*** | 2,480.22*** | |
|  | (123.46) | (0.01) | (0.03) | (632.58) | (0.02) | (0.03) | (0.01) | (344.56) | |
| Household income (Per £1000) | 19.47*** | - | 0.00** | 321.38*** | - | 0.02*** | -0.01*** |  | |
|  | (4.75) |  | (0.00) | (24.61) |  | (0.00) | (0.000) |  | |
| Constant | 2,561.27* | 0.223*** | -0.06 | -20,694.05** | 0.06 | -2.81*** | 0.45*** | 7,057.47 | |
|  | (1,291.47) | (0.066) | (0.28) | (6,930.08) | (0.24) | (0.30) | (0.12) | (3,787.317) | |
|  |  |  |  |  |  |  |  |  | |
| Observations | 4,146 | 3,394 | 5,435 | 4,501 | 3,779 | 5,436 | 5,426 | 5,436 | |
| *R²* | .025 | .009 | .007 | .083 | .011 | .101 | .129 | .139 | |
| Standard errors in parentheses | | | | | | | | |  |
| *** *p* < .001, ** *p* < .01, * *p* < .05 | | | | | | | | |  |

**Web Appendix B - Income**

The relationship between cognitive ability and income has been studied at length in past research. Therefore, we did not focus on income as a dependent variable in the main manuscript. We report these analyses here in Table B1 and Figure B1. After controlling for indicators of childhood SES as well as gender, marital status, and household size at age 46, we find a positive association between cognitive ability and income – those with greater cognitive ability generally earn a higher income. The relationship appears to be characterised by diminishing returns at lower levels of cognitive ability and by an exponential trend at higher levels of cognitive ability.

**Table B1.** *OLS regression models for the relationship between cognitive ability and income*

|  |  |  |  |
| --- | --- | --- | --- |
|  | (1) | (2) | (3) |
| VARIABLES | Income (£)  (Age 46) | Income (£)  (Age 46) | Income (£)  (Age 46) |
|  |  |  |  |
| Cognitive ability | 7,575.84*** | 6,487.38*** | 4,669.04*** |
|  | (777.27) | (754.89) | (934.67) |
| Cognitive ability^2^ | 1,274.82** | 1,221.19** | 1,151.41* |
|  | (397.40) | (385.27) | (465.03) |
| Cognitive ability^3^ | 228.08 | 220.52 | 333.50 |
|  | (215.87) | (208.99) | (264.95) |
| Female |  | -3,318.82*** | -3,524.44** |
|  |  | (918.35) | (1,076.48) |
| Married |  | 14,645.07*** | 14,167.37*** |
|  |  | (1,052.64) | (1,229.62) |
| Household size |  | 2,640.95*** | 2,583.20*** |
|  |  | (393.60) | (463.97) |
| Father’s education |  |  | 395.01 |
|  |  |  | (304.28) |
| Mother’s education |  |  | -7.79 |
|  |  |  | (316.66) |
| Family income at age 10 |  |  | 3,866.98*** |
|  |  |  | (478.08) |
| Constant | 39,678.95*** | 24,004.82*** | 3,535.92 |
|  | (595.41) | (1,359.77) | (5,243.62) |
|  |  |  |  |
| Observations | 5,858 | 5,858 | 4,450 |
| *R²* | .046 | .107 | .116 |
| Standard errors in parentheses, *** *p* < .001, ** *p* < .01, * *p* < .05 | | | |

**Figure B1.** Household income across levels of cognitive ability


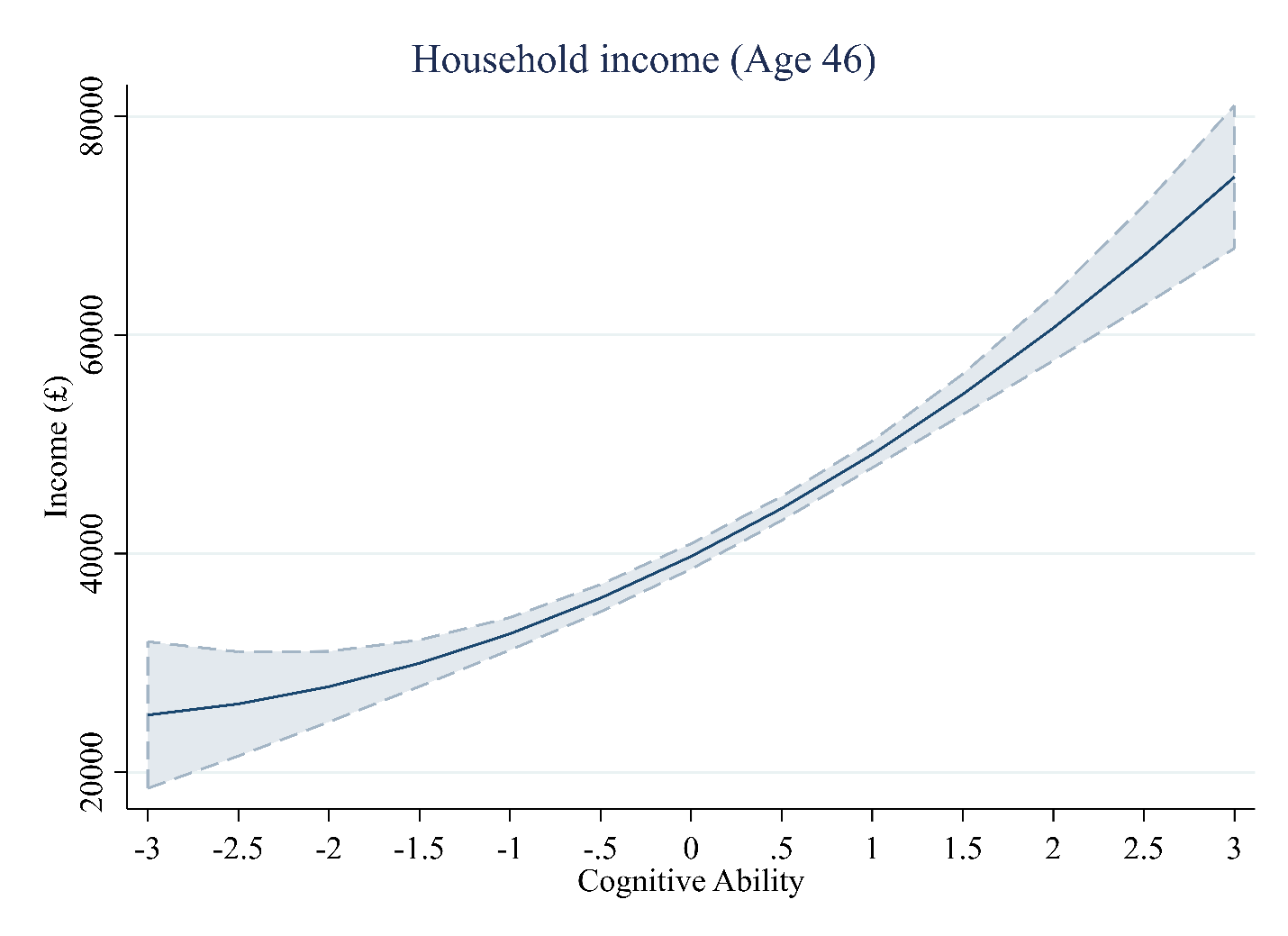


**Web Appendix C – Debt**

The relationship between debt and financial well-being may depend on the nature of the debt. Debt is a complex phenomenon that can have both positive and negative outcomes. In our manuscript, we argue that we focus on problematic debt by excluding credit card debt that is paid off monthly and by excluding mortgage debt. In this appendix, we further test the notion of whether our measure of debt is indeed indicative of problematic debt.

First, to strengthen the evidence that our debt measure reflects problematic debt, we conducted further regression analyses to examine whether debt predicts financial stress. Our findings indicate that debt is positively associated with financial stress in both the 2012 and 2016 waves, even after controlling for gender, marital status, household size, and income (Tables C1 and C2). We also found that these results remain significant when we include additional financial characteristics, such as assets and investments, as controls (Column 2 and 4). Moreover, our analyses revealed that total debt and credit card / store card debt are significant predictors of financial stress. These results suggest that debt is a negative financial outcome in our study.

**Table C1.** *OLS regression models for the relationship between debt and financial stress in the 2016 wave of the British Cohort Study*

|  |  | |  | |  | |  |
| --- | --- | --- | --- | --- | --- | --- | --- |
|  | (1) | | (2) | | (3) | | (4) |
| VARIABLES | Financial stress (2016) | | Financial stress (2016) | | Financial stress (2016) | | Financial stress (2016) |
|  |  | |  | |  | |  |
| Total debt  (per £1000) | 0.01*** | | 0.01*** | | - | | - |
|  | (0.00) | | (0.00) | |  | |  |
| Credit or store card debt |  | |  | | 0.27*** | | 0.25*** |
|  |  | |  | | (0.02) | | (0.02) |
| Female | 0.01 | | -0.01 | | -0.01 | | -0.03 |
|  | (0.02) | | (0.02) | | (0.02) | | (0.02) |
| Married | -0.31*** | | -0.25*** | | -0.32*** | | -0.25*** |
|  | (0.03) | | (0.03) | | (0.03) | | (0.03) |
| Household size | 0.04*** | | 0.05*** | | 0.04*** | | 0.05*** |
|  | (0.01) | | (0.01) | | (0.01) | | (0.01) |
| Household income (per £1000) | -0.01*** | | -0.01*** | | -0.01*** | | -0.01*** |
|  | (0.00) | | (0.00) | | (0.00) | | (0.00) |
| Household has investments |  | | -0.29*** | |  | | -0.29*** |
|  |  | | (0.02) | |  | | (0.02) |
| Total savings |  | | -0.00*** | |  | | -0.00*** |
|  |  | | (0.00) | |  | | (0.00) |
| Constant | 0.33*** | | 0.25*** | | 0.30*** | | 0.22*** |
|  | (0.03) | | (0.03) | | (0.03) | | (0.03) |
|  |  | |  | |  | |  |
| Observations | 7,187 | | 6,246 | | 7,322 | | 6,341 |
| *R²* | 0.144 | | 0.165 | | 0.145 | | 0.168 |
| Standard errors in parentheses | |  | |  | |  | |
| *** p<0.001, ** p<0.01, * p<0.05 | |  | |  | |  | |
|  | |  | |  | |  | |
|  | |  | |  | |  | |

**Table C2.** *OLS regression models for the relationship between debt and financial stress in the 2012 wave of the British Cohort Study*

|  |  |  |  |  |
| --- | --- | --- | --- | --- |
|  | (1) | (2) | (3) | (4) |
| VARIABLES | Financial stress (2012) | Financial stress (2012) | Financial stress (2012) | Financial stress (2012) |
|  |  |  |  |  |
| Total debt  (per £1000) | 0.02*** | 0.01*** | - | - |
|  | (0.00) | (0.00) |  |  |
| Credit or store card debt |  |  | 0.25*** | 0.18*** |
|  |  |  | (0.02) | (0.02) |
| Female | -0.06** | -0.07** | -0.07*** | -0.07** |
|  | (0.02) | (0.02) | (0.02) | (0.02) |
| Married | -0.22*** | -0.16*** | -0.24*** | -0.18*** |
|  | (0.03) | (0.03) | (0.02) | (0.02) |
| Household size | 0.04*** | 0.05*** | 0.05*** | 0.05*** |
|  | (0.01) | (0.01) | (0.01) | (0.01) |
| Household income (per £1000) | -0.01*** | -0.01*** | -0.01*** | -0.01*** |
|  | (0.00) | (0.00) | (0.00) | (0.00) |
| Household has investments |  | -0.24*** |  | -0.27*** |
|  |  | (0.03) |  | (0.02) |
| Total savings |  | -0.00*** |  | -0.00*** |
|  |  | (0.00) |  | (0.00) |
| Constant | 0.25*** | 0.29*** | 0.26*** | 0.32*** |
|  | (0.03) | (0.04) | (0.03) | (0.03) |
|  |  |  |  |  |
| Observations | 6,818 | 5,679 | 9,049 | 7,422 |
| *R²* | 0.137 | 0.196 | 0.135 | 0.198 |
| Standard errors in parentheses |  |  |  |  |
| *** p<0.001, ** p<0.01, * p<0.05 |  |  |  |  |

Second, we test the robustness of our findings for the inverse U-shape relationship between cognitive ability and debt by taking a more strict classification of debt. In our study, we excluded sources of "good" debt, such as mortgages and credit card debt that will be paid off by the end of the month, to ensure that our measure of debt truly reflects problematic debt. However, one category of debt that may be considered "good" is student loans, which only 3.74% of respondents in our sample reported as a source of debt in the 2016 wave of the British Cohort Study. To further confirm that our findings are not driven by "good" debt, we conducted additional analyses excluding respondents who reported student loans as a source of debt. As reported in Table C3, we find that after excluding this group, we still observed an inverse U-shaped relationship between cognitive ability and total debt, and the cubic term of cognitive ability remains statistically significant.

**Table C3.** *OLS regression models for the relationship between cognitive ability and debt*

|  |  |  |  |  |
| --- | --- | --- | --- | --- |
|  | (1) | (2) | (3) | (4) |
| VARIABLES | Total debt 2016 | Total debt 2016 | Total debt 2016 | Total debt 2016 |
| Cognitive ability |  |  |  |  |
|  | 844.55*** | 756.97*** | 689.06** | 515.56* |
| Cognitive ability^2^ | (186.27) | (185.42) | (230.19) | (241.36) |
|  | -404.86*** | -432.17*** | -457.45*** | -493.15*** |
| Cognitive ability^3^ | (95.93) | (95.31) | (115.01) | (120.69) |
|  | -106.93* | -114.79* | -107.35 | -104.69 |
| Female | (52.22) | (51.81) | (65.90) | (68.29) |
|  |  | -1,174.50*** | -1,369.47*** | -1,243.70*** |
| Married |  | (227.15) | (266.56) | (278.66) |
|  |  | 1,059.12*** | 1,280.55*** | 969.96** |
| Household size |  | (259.46) | (303.41) | (322.83) |
|  |  | 512.47*** | 454.40*** | 394.05** |
| Father’s education |  | (97.22) | (114.66) | (120.25) |
|  |  |  | -51.03 | -60.19 |
| Mother’s education |  |  | (75.31) | (80.51) |
|  |  |  | 163.18* | 179.96* |
| Family income at age 10 |  |  | (78.63) | (82.99) |
| Cognitive ability |  |  | 57.28 | -49.37 |
|  |  |  | (118.43) | (124.84) |
| Household income (per £1000) |  |  |  | 25.98*** |
|  |  |  |  | (3.84) |
| Constant | 5,121.05*** | 3,473.40*** | 1,753.07 | 1,508.59 |
|  | (143.73) | (336.16) | (1,286.72) | (1,364.77) |
|  |  |  |  |  |
| Observations | 5,937 | 5,937 | 4,510 | 4,221 |
| *R^2^* | .007 | .023 | .025 | .036 |
| Standard errors in parentheses |  |  |  |  |
| *** p<0.001, ** p<0.01, * p<0.05 |  |  |  |  |

**Figure C1.** Total debt across levels of cognitive ability, excluding student debt


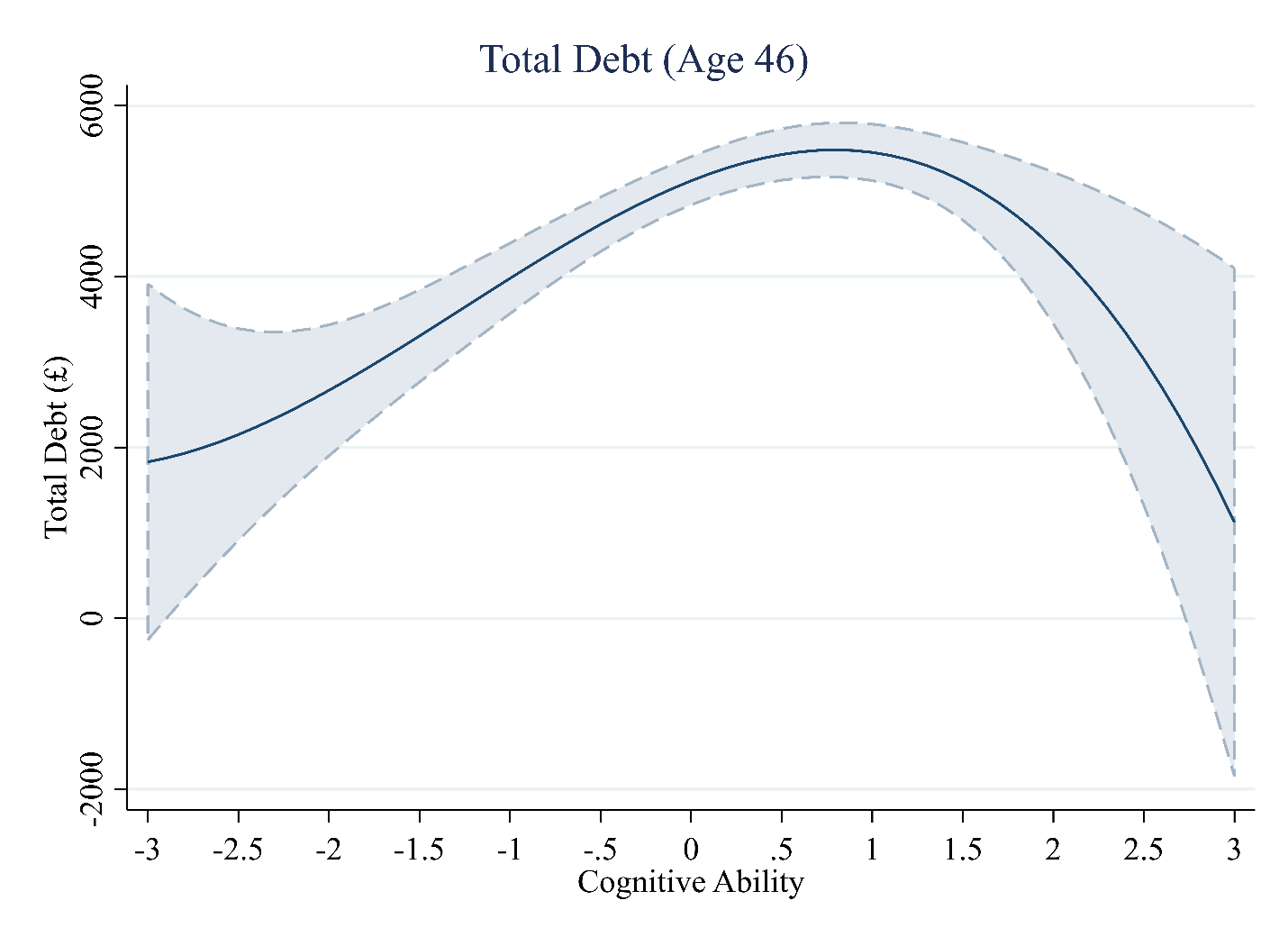

Supplement: S1 File — (DOCX) [file pone.0285199.s001.docx]
